# Expression analyses in *Ginkgo biloba* provide new insights into the evolution and development of the seed

**DOI:** 10.1038/s41598-021-01483-0

**Published:** 2021-11-09

**Authors:** Cecilia Zumajo-Cardona, Damon P. Little, Dennis Stevenson, Barbara A. Ambrose

**Affiliations:** 1 New York Botanical Garden, Bronx, NY USA; 2 The Graduate Center, City University of New York, New York, NY USA

**Keywords:** Developmental biology, Evolution, Plant sciences

## Abstract

Although the seed is a key morphological innovation, its origin remains unknown and molecular data outside angiosperms is still limited. *Ginkgo biloba,* with a unique place in plant evolution, being one of the first extant gymnosperms where seeds evolved, can testify to the evolution and development of the seed. Initially, to better understand the development of the ovules in *Ginkgo biloba* ovules, we performed spatio-temporal expression analyses in seeds at early developing stages, of six candidate gene homologues known in angiosperms: *WUSCHEL, AINTEGUMENTA, BELL1, KANADI, UNICORN,* and *C3HDZip*. Surprisingly, the expression patterns of most these ovule homologues indicate that they are not wholly conserved between angiosperms and *Ginkgo biloba*. Consistent with previous studies on early diverging seedless plant lineages, ferns, lycophytes, and bryophytes, many of these candidate genes are mainly expressed in mega- and micro-sporangia. Through in-depth comparative transcriptome analyses of *Ginkgo biloba* developing ovules, pollen cones, and megagametophytes we have been able to identify novel genes, likely involved in ovule development. Finally, our expression analyses support the synangial or neo-synangial hypotheses for the origin of the seed, where the sporangium developmental network was likely co-opted and restricted during integument evolution.

## Introduction

The seed, critical for the successful evolution and diversification of plants, is the salient synapomorphy of seed plants, but its origin and relationships amongst extant seed plant lineages remains unclear. The seed develops from an ovule that is composed of a megasporangium, conserved in land plants, covered by the integument(s), the defining step in seed evolution^1^. Historically, the evolution of the integuments, and therefore of the seed, is a subject that has aroused great interest from scientists, which has led to various proposals, including three major hypotheses that remain valid and which all have supporting paleontological and morphological evidence. (1) The “de novo” hypothesis, that states that the integument covering the sporangia appeared as a new structure^2,3^. (2) The “telome” hypothesis, supported by the fusion of integumentary lobes in the Palaeozoic ovules, suggesting that integuments are the result of the fusion of vegetative structures, telomes, around the sporangium^4,5^. (3) The “synangial” hypothesis, that proposes that integuments are the result of sterilization of sporangia around the only sporangium that remains functional^6–8^. Later, following evidence of the vascular traces of the Palaeozoic ovules, the synangial hypothesis was modified^9^, evidence that led to the ‘neo-synangial” hypothesis. Recent studies on anatomical development of ovules in Cycadales and the fossil record of *Genomosperma kidstonii*^10,11^, as well as molecular studies from *Arabidopsis thaliana* (*Arabidopsis*^12^) and *Gnetum gnemon* (*Gnetum*^13^) support the neo-synangial hypothesis.

The molecular mechanisms underlying seed development are widely known in angiosperms but for gymnosperms, data are rather scarce. Comparative molecular analyses of integument development between angiosperms and *Ginkgo biloba* (*Ginkgo*), provide essential data to better understand the origin and evolution of the seed. Known as a living fossil, the gymnosperm *Ginkgo* has remained morphologically unchanged since it evolved nearly 300 mya^14^ and constitutes one of the first extant plant lineages where seeds evolved^14^.

In *Arabidopsis thaliana*, three transcription factors are known to play an essential role in the initiation of the integuments by different mechanisms, *AINTEGUMENTA* (*ANT*), *WUSCHEL* (*WUS*) and *BELL1* (*BEL1*)^15–18^. *WUS* in *Arabidopsis*, is required for the proper establishment of the chalaza promoting formation of the integuments. In fact, *wus* mutants do not develop integuments while overexpression of *WUS* results in supernumerary integuments^16,17^. Moreover, the expression of *WUS* in *Arabidopsis* is restricted to the nucellus activating a downstream signal derived from the nucellus, inducing organ initiation in the adjacent chalaza cells; *WUS* forms a short-range signaling module repeatedly during plant development^16^. *WUS* in *Arabidopsis* also regulates cell differentiation in anther development, and it is expressed in the pollen^19^. *WUS* function in the maintenance of stem cells appears to be conserved in core eudicots but not in monocots where it is essential in axillary meristem initiation^20–24^.

In angiosperms, *ANT* homologues act in the development of the two integuments, as well as in the control of leaf size^25,26^. The *ant* mutant in *Arabidopsis*, has smaller leaves, fewer floral organs, lacks integuments and megasporogenesis is blocked^15,27–29^. These pleiotropic roles of *ANT* in plant development are the result of its control over cell proliferation^15^. In angiosperm ovules, *BEL1* homologues are restricted to the integument, and this pattern is conserved across angiosperms^30,31^. In *Arabidopsis*, this function, seems to be due to the interaction of *BEL1* with the carpel identity dimer *AGAMOUS-SEPATALLATA3* and to the repression of *WUS* towards the nucellus^24^. Another suggested genetic interaction, possibly related to BEL1 function in integument formation, is the repression of *SPOROCYTELESS/NOZZLE* (*SPL/NZZ*), a master regulator of nucellus-forming pathways upregulating PIN-FORMED 1 (PIN1) and WUS^17,32^.

Once integument development has started, multiple transcription factors come into play for the patterning of the integuments including *Class III HD-Zip*s (*C3HDZ* or *C3HD-Zip*), *KANADI*s (*KAN*s), and *UNICORN* (*UCN*). There are five *Arabidopsis Class III HD–Zip* genes: *AtHB8*, *CORONA/AtHB15* (*CNA*), *PHABULOSA* (*PHB*), *PHAVOLUTA* (*PHV*), and *REVOLUTA* (*REV*)^33–35^; that are well known for their role in establishing the adaxial side of the leaf^35^. *CNA*, *PHB*, *PHV* and *REV* have been reported to be involved in the proper establishment of planar polarity of the integument, where they are expressed adaxially; and *CNA*, *PHB* and *PHV* are restricted to the inner integument^17,36–39^.

In *Arabidopsis*, *KANs* are responsible for specifying the abaxial identity of leaves and integument. *KAN1* and *KAN2*, play a role in abaxial identity of the outer integument^40–43^. *ABERRANT TESTA SHAPE* (*ATS*) also known as *KANADI 4* (*KAN4*)*,* specifies the abaxial identity of the inner integument^44^. As for integument polarity, their function seems to be conserved across angiosperms as the same patterns are observed in the early diverging angiosperm, *Amborella trichopoda*^45^. In *Arabidopsis*, *UCN* is involved in the planar identity of the outer integument by controlling cell growth and repressing *KAN4*^46^.

Phylogenetic analyses in land plants show that each of these genes has undergone multiple independent duplication events^47–50^. In gymnosperms, these genes have been studied in *Gnetum* species, *GgWUS,* is expressed in the nucellus, similarly to angiosperms (Nardmann et al.^48^). *ANT*, *GneANT*, is expressed in the integument as well as in the nucellus; *Melbel1*, *BEL1* homolog in *Gnetum* is restricted to the nucellus^13^. Interestingly, the *KAN* and *UCN* homologs are mainly restricted to the apical portion of the *Gnetum* integument^13^. Our results of spatiotemporal expression analyses for *WUS*, *ANT*, *BEL1*, *KANs*, *Class III HD-Zips*, and *UCN* homologues in *Ginkgo* show that changes in their expression patterns in seed plants, may be linked to major developmental differences. The transcriptome analyses we performed to identify differentially expressed genes, revealed putative candidate genes for *Ginkgo* integument development. One of these candidate genes is *FANTASTIC FOUR* (*FAF*), a plant-specific gene family, characterized in *Arabidopsis* for its role in meristem development and its interaction with WUS^51^. In *Ginkgo*, expression of *FAF* is restricted to the integument, suggesting a role in *Ginkgo* ovule development. Moreover, the results from the expression analyses provide molecular evidence supporting the hypotheses that the ovule evolved from sterilization and fusion of sporangia^6,9^.

## Results

### Expression analyses of *WUSCHEL* homologues in *Ginkgo*: *GbWUS*

**Figure 1:**
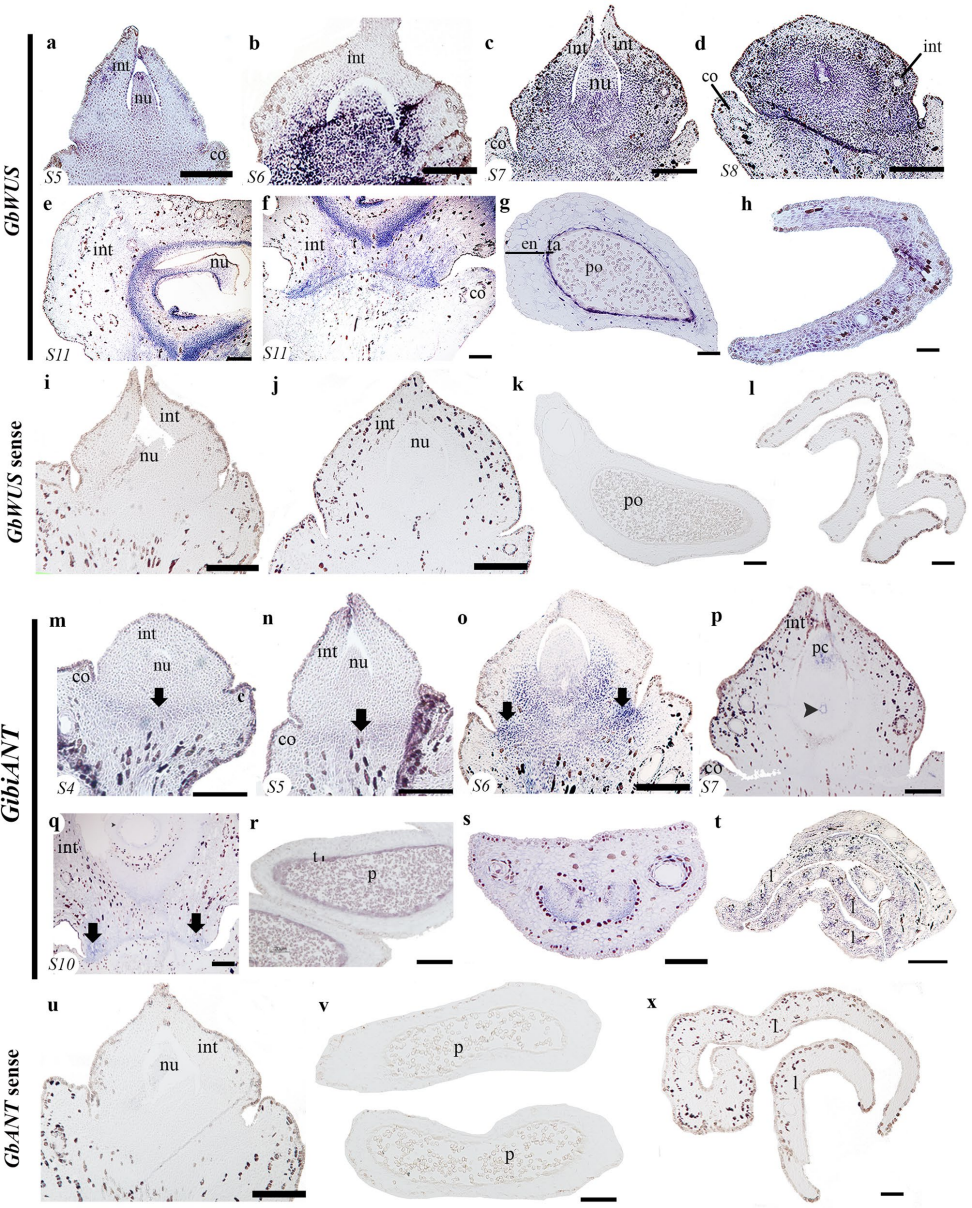
Expression of *GibiWUS* and *GibiANT* using in situ hybridization. (**a–h)** *GibiWUS* expression patterns. **(a)** Ovule in stage 5 (S5). **(b)** Ovule in stage 6 (S6). **(c)** Ovule in stage 7 (S7), pollination stage. **(d)** Ovule in stage 8 (S8). **(e)** Ovule in stage 11 (S11). **(f)** Close-up of the abscission zone of the same ovule at stage 11 (S11). **(g)** Expression in the pollen cone. **(h)** Cross section of a leaf. **(i–l**) *GbWUS* sense probe. (**m–t)** *GibiANT* expression patterns. **(m)** Ovule in stage 4 (S4). **(n)** Ovule in stage 5 (S5). **(o)** Ovule in stage 6 (S6). **(p)** Ovule in stage 7 (S7). **(q)** Ovule in stage 10 (S10). **(r)** Microsporangium. **(s)** Petiole of the leaf. **(t)** Cross section of the leaf. (**u–x**) *GbANT* sense prone. The corresponding stage (S) is shown at the bottom left of each picture. *Black arrows* pointing to the abscission zone; *black arrowheads* pointing to the megaspore mother cell*; co* collar, *en* endothelium, *int* integument, *nu* nucellus, *po* pollen, *ta* tapetum. Scales: 50 μm (**a,e–n,q,r**); 75 μm (**b,k,l,s,t**); 100 μm (**c,d**).

The development of the *Ginkgo* ovule has been divided into 11 stages (stage = S; Fig. S1)^52^. When the integument overtops the nucellus (S5), *GbWUS* is strongly expressed in the nucellus and in a layer of cells known as the abscission zone of the ovule, that is between the ovule base and the collar, a region from which the ovule will detach from the plant when the seed is fully mature (Fig. 1a). Its expression is also detected in the integument, which already covers the nucellus (Fig. 1a). During S6, before the ovule is fertilized, *GbWUS* is strongly expressed in the nucellus and the base (proximal region) of the integument (known as pachychalazal region) as well as in the abscission zone (Fig. 1b). At pre-pollination S7, *GbWUS* expression is maintained in the proximal region of the nucellus, and the integument (in the pachychalaza region). No *GbWUS* expression is detected in the apical region of the integument, which forms the micropyle (Fig. 1c). These expression patterns are maintained as the ovule matures to S8. However, no expression is detected in the collar (Fig. 1d). Later on, in S11, *GbWUS* expression is detected in the inner side of the integument corresponding to the endotesta, nucellus, jacket cells and in the proximal region of the ovule in the abscission zone (Fig. 1e,f). *GbWUS* is also expressed in nearly mature pollen grains and the tapetum (Fig. 1g) and also in the young but well-developed leaves (Fig. 1h). No signal was detected with a *GbWUS* sense probe (Fig. 1i–l).

### Expression analyses of *ANT Ginkgo* homologues*: GibiANT*

*GibiANT* expression was consistent throughout ovule development. In S4 and S5 of ovule development, the expression of *GibiANT* is limited to the region which will become the abscission zone of the ovule (Fig. 1m,n). It is also found at the distal end of the integument, which will form the micropyle (Fig. 1n). When the development of the megaspore mother cell begins, at S6, *GibiANT* is expressed in the chalazal region, and particularly in the abscission zone towards the region close to the collar (Fig. 1o). In S7, once the nucellus and the megaspore mother cell are well formed, the expression of *GibiANT* is also detected in the jacket cells and in the pollen chamber (Fig. 1p). Later, in S10, *GibiANT* expression is maintained in the abscission zone laterally, close to the collar (Fig. 1q). *GibiANT* is expressed in the tapetum of the pollen cone and in the nearly mature pollen grains (Fig. 1r). Furthermore, *GibiANT* has been found widely expressed in the vegetative tissue, in the petiole of the leaf, the young leaves and the vascular bundles (Fig. 1s,t). No signal was detected with a *GibiANT* sense probe (Fig. 1u–x).

### Expression analyses of *BELL1 Ginkgo* homologues: *GibiBEL1* and *GibiBEL1-2*

**Figure 2:**
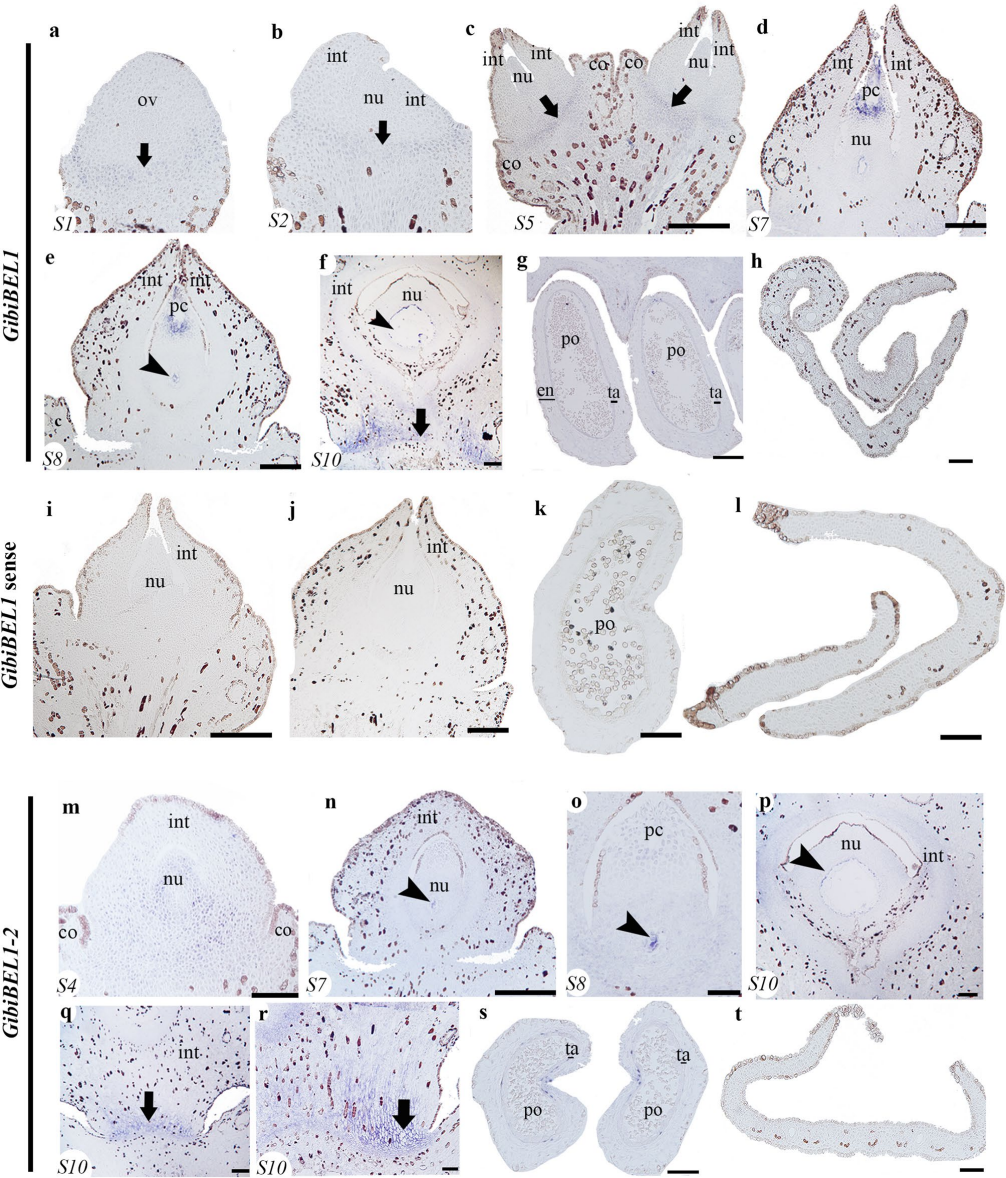
Expression of *BEL1* homologues using in situ hybridization. **(a–h)** Expression patterns of *GibiBEL1*. **(a)** Ovule in stage 1 (S1). **(b)** Ovule in stage 2 (S2). **(c)** Ovule in stage 5 (S5). **(d)** Ovule in stage 7 (S7). **(e)** Ovule in stage 8 (S8). **(f)** Stage 10 (S10), close-up of the abscission zone. **(g)** Pollen cone. **(h)** Cross section of the leaf. **(i–l)** *GibiBEL1* sense probe as control. **(m–t)** Expression patterns of *GibiBEL1*-2. **(m)** Ovule in stage 4 (S4). **(n)** Ovule in stage 7 (S7). (**o)** Close-up of the nucellus of an ovule in stage 8 (S8). **(p)** Close-up of the nucellus of an ovule at stage 10 (S10). **(q,r)** Abscission zone of the ovule in stage 10 (S10). **(s)** Microsporangia. **(t)** Cross section of a leaf. The corresponding ovule stage (S) is shown at the bottom left of each picture. *Black arrows* pointing to the abscission zone; *black arrowheads* pointing to the megaspore mother cell; *co* collar, *en* endothelium, *int* integument, *nu* nucellus, *po* pollen, *ta* tapetum. Scales: 50 μm (**c,d,i,j,l,r,t**); 75 μm (**e,k,n,o**); 100 μm (**f–h,p–s**).

Expression analyses of the two *BELL1* homologues, *GibiBEL1* and *GibiBEL1-2,* in developing ovules show restricted expression patterns for each copy. At S1 and S2, *GibiBEL1* is expressed at the base of the ovule (Fig. 2a,b). At S5, *GibiBEL1* is expressed in the abscission zone (Fig. 2c). At S7, when the ovule is competent for fertilization, *GibiBEL1* is detected in the pollen chamber (Fig. 2c) and at S8, in the megaspore mother cell and jacket cells once they have formed (Fig. 2d,e). At S10, *GibiBEL1* is strongly expressed in the abscission zone, and detected in the nucellus and endotesta cells of the integument (Fig. 2f). No *GibiBEL1* expression was found in the young developing pollen cone or in the blade of the young leaf (Fig. 2g,h). No signal was detected with a *GibiBEL1* sense probes (Fig. 2i–l).

Unlike *GibiBEL1*, *GibiBEL1-2* is expressed in the nucellus from the early stages of ovule development (S4; Fig. 2m) with this expression restricted to the megaspore mother cell, once it develops (S7–8; Fig. 2n,o). *GibiBEL1-2* is also expressed in the jacket cells (S10, Fig. 2p) and in the abscission zone (Fig. 2q,r). No expression was detected in the pollen cones or the leaves (Fig. 2s.t).

### Expression analyses of *Ginkgo* homologues *GibiKAN, GibiUCN, GibiUCN2* and *GbC3HDZs*

**Figure 3:**
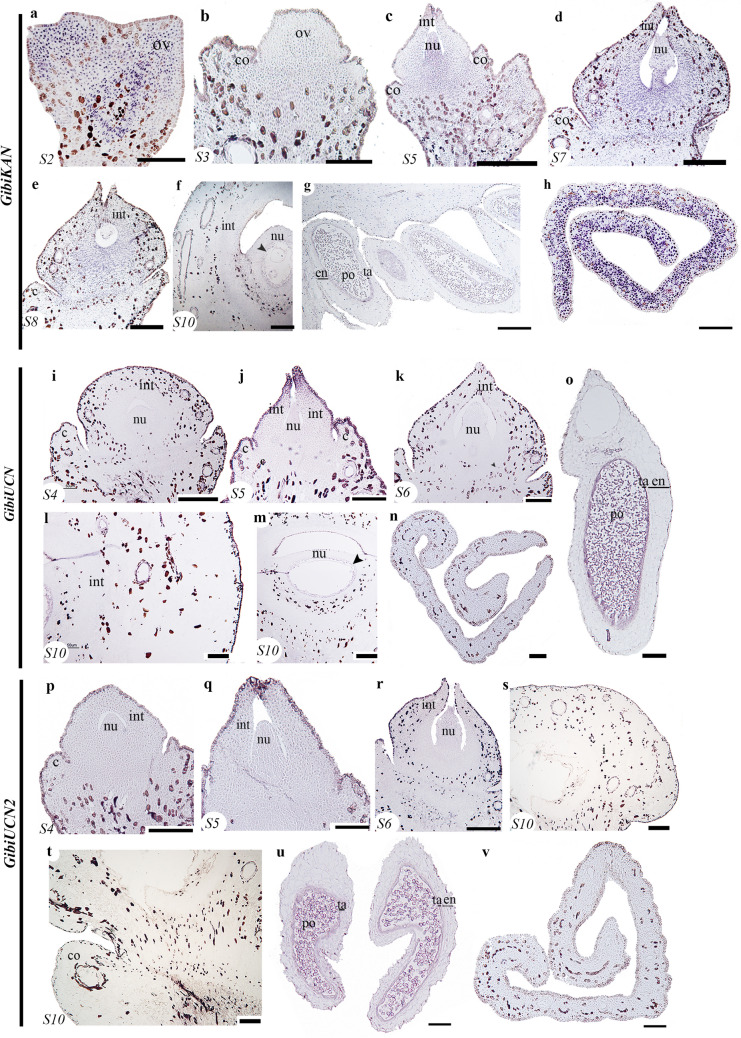
Expression patterns of *GibiKAN*, *GibiUCN* and *GibiUCN2* using in situ hybridization. **(a–h)** *GibiKAN* expression patterns. **(a)** Ovule in stage 2 (S2). **(b)** Ovule in stage 3 (S3). **(c)** Ovule in stage 5 (S5). **(d)** Ovule in stage 7 (S7). (**e)** ovule in stage 8 (S8). **(f)** Ovule in stage 10 (S10). **(g)** Pollen cone. **(h)** Cross section of the leaf. **(i–o)** *GibiUCN* expression patterns. **(i)** Ovule in stage 4 (S4). **(j)** Ovule in stage 5 (S5). **(k)** Ovule in stage 6 (S6). **(l)** Integument of an ovule in stage 10 (S10). (**m)** Megagametophyte of an ovule in stage 10 (S10). **(n)** Cross section of a leaf. **(o)** Microsporangium. **(p–v)** *GibiUCN2* expression patterns. **(p)** Ovule in stage 4 (S4). **(q)** Ovule in stage 5 (S5). **(r)** Ovule in stage 6 (S6). **(s,t)** Ovule in stage 10 (S10). **(u)** Microsporangium. **(v)** Cross section of the leaf. The corresponding ovule stage (S) is shown at the bottom left of each picture. *co* collar, *en* endothelium, *int* integument, *nu* nucellus, *po* pollen, *ta* tapetum. Scales: 50 μm (**a,b,f,i,l–n,p,s–v**); 75 μm (**c–e,q,r**); 100 μm (**g,j,k,o**).

Initially, in S2, *GibiKAN* is expressed throughout the ovule primordia and the funiculus (Fig. 3a). Later, at S3, *GibiKAN* is expressed in the region that will become the nucellus (Fig. 3b) and it is maintained as the nucellus develops, 5 (Fig. 3c). At this stage *GibiKAN* is also expressed in the apical region of the integument (Fig. 3c). In S7 and 8, *GibiKAN* is also expressed in the integument when the integument begins to close the micropyle (Fig. 3d,e). These expression patterns in the integument and nucellus are maintained, and additional expression is detected in the jacket cells at S10 (Fig. 3f). *GibiKAN* is also expressed in microspores and pollen grains (Fig. 3g). In vegetative tissues, *GibiKAN* is highly expressed throughout leaf development and its expression does not appear polar (Fig. 3h). Sense probes show no expression (Fig. S10).

The two *UNICORN* homologues, *GibiUCN* and *GibiUCN2,* show low levels of expression throughout ovule development (Fig. 3i–t). As the integument becomes distinct from the nucellus, *GibiUCN* is specifically expressed in the apical region of the integument forming the micropyle (Fig. 3j). Both paralogues are strongly expressed in the tapetum and in the nearly mature pollen grains (Fig. 3o–u). We did not detect expression of either homologue in the blade of young leaves (Fig. 3n,v). Sense probes show no expression (Fig. S10).

**Figure 4:**
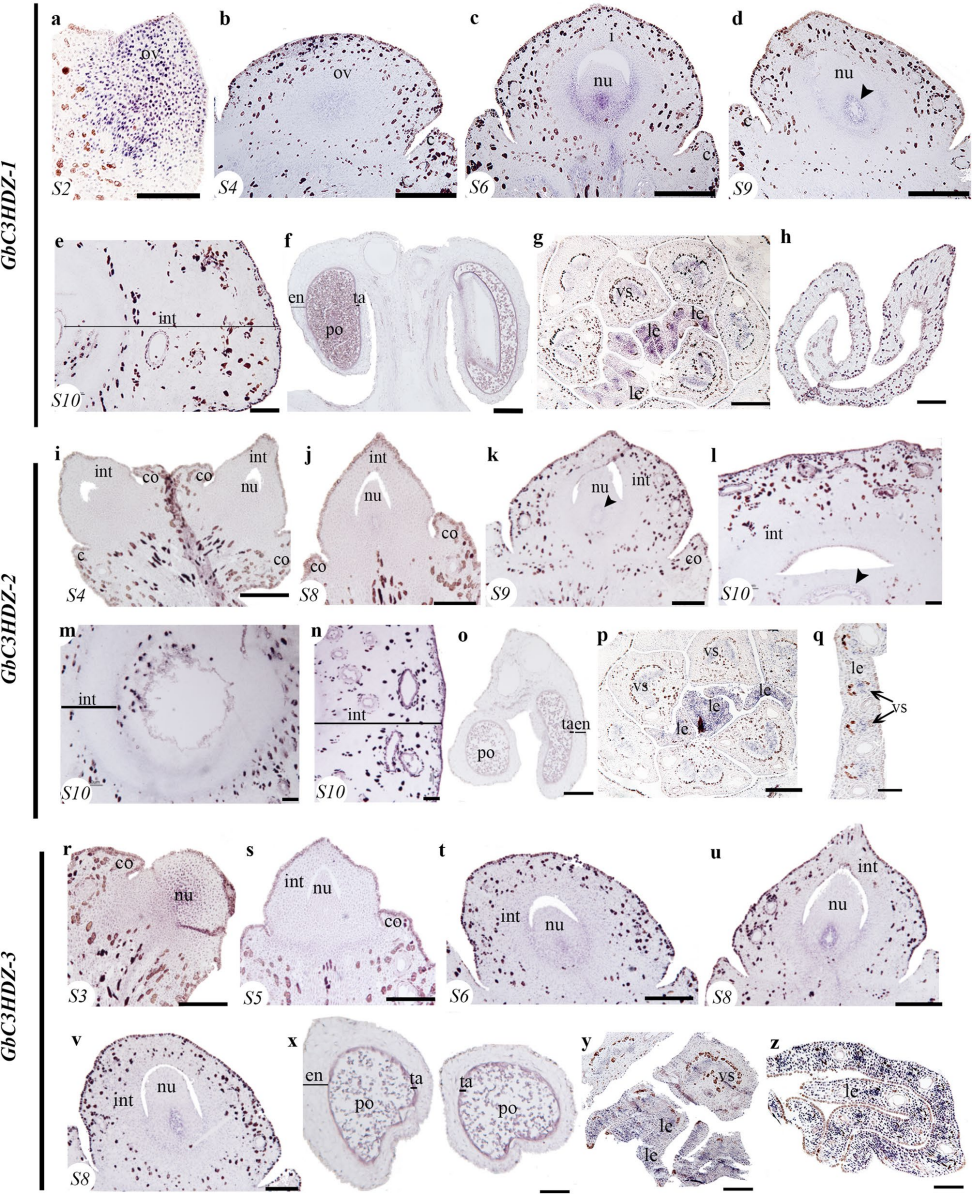
Expression patterns of three *C3HDZ*s homologues. **(a–h)** Expression patterns of *GbC3HDZ-1*. **(a)** Ovule in stage 2 (S2). **(b)** Ovule in stage 4 (S4). **(c)** Ovule in stage 6 (S6). **(d)** Ovule in stage 9 (S9). **(e)** Integument of an ovule in stage 10 (S10). **(f)** Microsporangium, showing expression in the pollen grains and tapetum. **(g)** Cross section of a short shoot with leaf primordia in the center. **(h)** Cross section of a well-developed leaf. **(i–q)** Expression patterns *GbC3HDZ-2.* **(i)** Ovules in stage 4 (S4). **(j)** Ovule in stage 8 (S8). **(k)** Ovule in stage 9 (S9). **(l,m)** Ovule in stage 10 (S10). **(o)** Microsporangia. **(p)** Cross section of a short shoot with leaf primordia in the center. **(q)** Cross section of a well-developed leaf. **(r–z)** Expression patterns *GbC3HDZ-3.* **(r)** Ovule in stage 3 (S3). **(s)** Ovule in stage 5 (S5). **(t)** Ovule in stage 6 (S6). **(u)** Ovule in stage 8 (S8). **(v)** Ovule in stage 9 (S9). **(x)** Microsporangia. **(y)** Cross section of a short shoot with leaf primordia in the center. **(z)** Cross section of a leaf. *Black arrowheads* pointing to the megaspore mother cell; *co* collar, *en* endothelium, *int* integument, *le* leaf, *nu* nucellus, *po* pollen, *ta* tapetum, *vs* vasculature. Scales: 50 μm (**a,b,i,j,r–t**); 75 μm (**c,e–g,k,p,u,v,y**); 100 μm (**d,h,l–o,q,x,z**).

**Figure 5:**
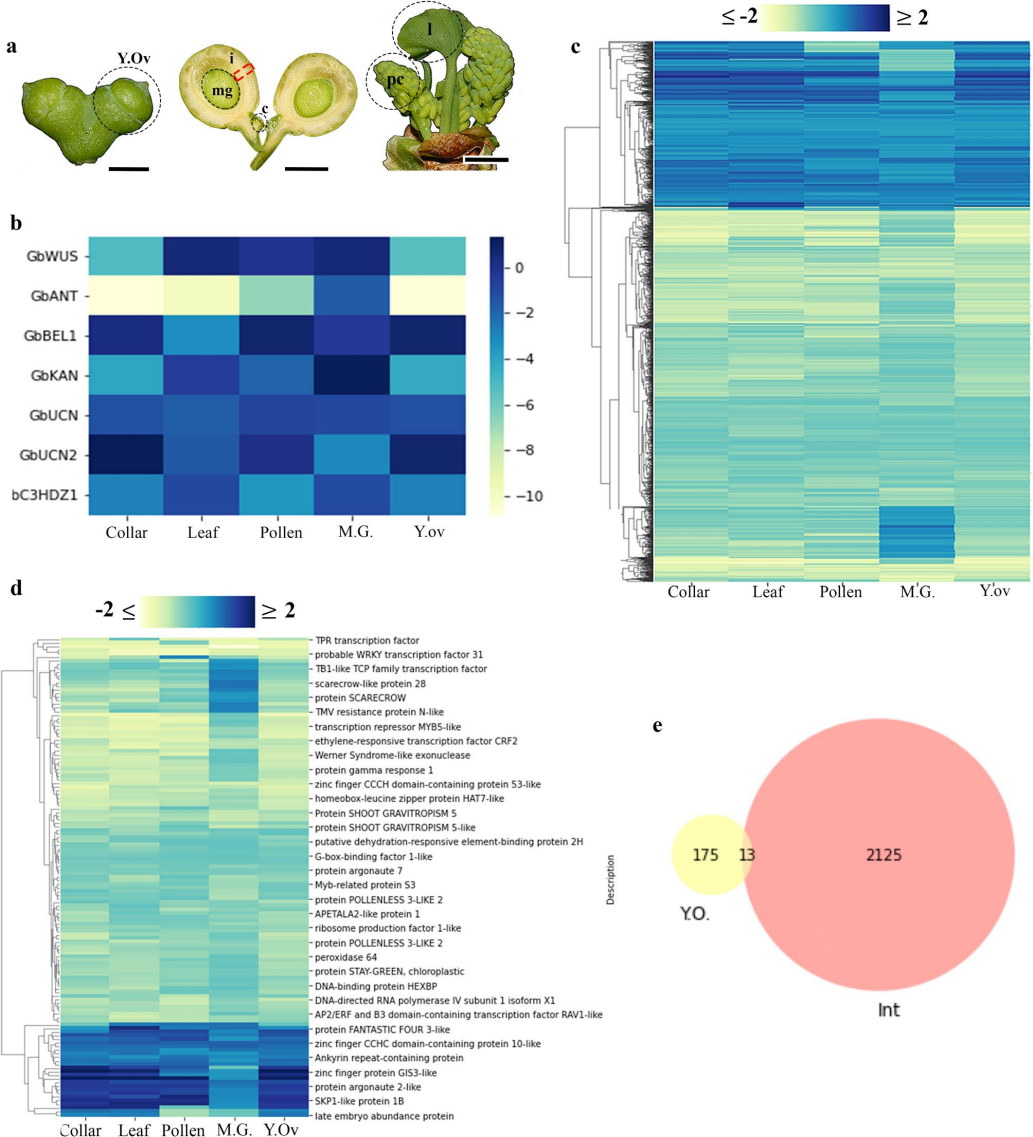
Transcriptome results focused on integument development. **(a)** *Ginkgo* samples that were sequenced separately to perform the differential expression analyses. Red square indicates the integument. **(b)** Heatmap of the genes from the integument developmental network differentially expressed in the integument. **(c)** Cluster map throughout all the tissues compared to the integument, 2137 Differentially Expressed (DE) genes with a fold expression change between − 2 and 2 and good transcriptional support (TPM ≥ 0.95). Each column of the cluster map indicated the twofold changes of each sample with respect to the integument. **(d)** 134 DE transcription factors differentially expressed in the integument compared to all the other samples. Two clusters were identified that largely consisted of up-regulated (blue clusters, n = 21) and down-regulated genes (yellow, n = 97). **(e)** Comparison of the DE genes between the young ovule sample and the integument. **(f)** Comparison of the DE genes between the megagametophyte and the integument. *Blue*, upregulated genes and *yellow*, downregulated genes (**b–d**).

From the five paralogues identified for the *C3HDZip* genes in *Ginkgo, GbC3HDZ1* to *5*^49^, we were able to assess the expression of four paralogs *GbC3HDZ1* to* 4* (Fig. 4, Supplementary Fig. S11)*.* At S2, *GbC3HDZ1* is expressed in the ovule primordia (Fig. 5a); and in S4 and S6, in the young developing nucellus (Fig. 4b,c) specifically in the region where the megaspore will develop (Fig. 4c). *GbC3HDZ1* is expressed in the adaxial side of the integument, in the region that delimits the integument and nucellus (Fig. 4c). This expression is maintained in the adaxial side of the integument and in the jacket cells, S9 (Fig. 4d). At S10, in the fleshy integument, we did not detect expression of *GbC3HDZ1* (Fig. 4e), but it is expressed in the tapetum of the microsporangium and in the nearly mature pollen grains (Fig. 4f). *GbC3HDZ1* is detected in the leaf and petiole vasculature and appears adaxial in young developing leaves (Fig. 4g). *GbC3HDZ1* is not detected in the blade of well-developed leaves (Fig. 4h). *GbC3HDZ1* sense probes show no expression (Fig. S10).

In ovules at S4, no expression of *GbC3HDZ2* was detected (Fig. 4i) but in S8, as soon as the megaspore and the jacket cells start to develop, expression is detected (Fig. 4j). This expression is maintained as the ovule matures, S9 (Fig. 4k). Later, at S10 after pollination, *GbC3DZ2* is found expressed in the jacket cells (Fig. 4l,m) and throughout the fleshy seed coat (Fig. 4n). *GbC3DZ2* expression is detected in the tapetum and the nearly mature pollen grains (Fig. 4o) and in young developing leaves (Fig. 4p) becoming restricted to the vascular bundles and the adaxial side of the well-developed leaves (Fig. 4q).

*GbC3HDZ3* is expressed similarly to *GbC3HDZ1* in the young developing nucellus (Fig. 4r), the jacket cells, the tissue that will form the megaspore, the adaxial side of the integument in the region in close contact with the nucellus (Fig. 4t–v), in the tapetum and pollen grains (Fig. 4x), and throughout the vegetative tissue including vascular bundles (Fig. 4y,z). *GbC3HDZ4* is expressed at S11 in well-developed ovules in the inner region of the integument, the endotesta (Fig. S11a–e) and the pollen grains (Fig. S11f). No expression of *GbC3HDZ4* is detected in the leaf (Fig. S11g).

### Transcriptome assembly

A de novo reference transcriptome of *Ginkgo* was generated from RNAs isolated from young ovule (S4), integument, megagametophyte, collar (dissected from ovules in S9), pollen cone and leaf. Using Trinity software 86,050 transcripts were obtained, with an average GC content of 41.52% and a maximum assembled contig length of 18,726 bases. To improve the quality of the assembly, the contigs were mapped to the initial assembly with ABySS. This gives a final total of 53,970 transcripts (Table 1). Based on read coverage, the E90N50 statistic was ~ 1.8 Kb (Fig. S6), the reference transcriptome contained 86.9% of the conserved Embryophyte genes using BUSCO annotation (Fig. S7).

**Table 1:** Statistics for *Ginkgo* reference transcriptome.

Parameter	Number
***Ginkgo* reference transcriptome**	
Total trinity transcripts	86,050
Total trinity ‘genes’	46,636
%GC	41.52
Longest contig (bp)	18,726
shortest contig	201
Number of contigs > 200 bp	86,050
Number of contigs > 1 Kb	46,316
Number of contigs > 5 kb	2488
Number of contigs > 10 Kb	117
Number of predict ORFs (transdecoder)	67,040
**Stats after re-assembly with AbySS**	
Total transcripts after re-assembly AbySS	53,970
Contigs longer than 200	36,979
Contigs longer than 1 kb	14,685
Contigs longer than 5 kb	364
Contigs longer than 10 kb	17
Number of predict ORFs (transdecoder)	36,979

The initial assembly was improved with a re-assembly method using AbySS.

Samples were compared with a Principal Component Analysis (PCA), which shows that the integument and the megagametophyte are the most dissimilar samples in the data set in terms of gene expression (Fig. S8a). A hierarchical cluster analysis was performed to better understand the similarities within samples. The resulting dendrogram shows that the integument is the most distinct sample with longer branch distance (Y-axis) but it is more similar to the megagametophyte (Fig. S9). Collar, leaf, pollen cone, and young ovules form another cluster (Fig. S9).

### Differentially expressed genes in the integument of *Ginkgo*

To identify genes that are differentially expressed (DE) in the integument of *Ginkgo*, transcriptome analyses were performed in different plant tissues (i.e., young ovules, integument, megagametophyte, collar, pollen cone, and leaf; Fig. 5a, Table S1). Differentially expressed genes in the integument were filtered by statistical significance (FDR p ≤ 0.05) and a comparison of all tissues against integument was performed. We found that most of the DE genes, that belong to the ovule genetic network seem to be similarly upregulated in all tissues except for *GibiANT* (Fig. 5b). Subsequently, to focus on genes with a larger change (log2FC ≤  − 2 and ≥ 2), we added a Fold Change threshold which detected 2137 DE genes (Fig. 5c). None of the genes in the ovule regulatory network passed this filter.

### Transcription factors differentially expressed in integument

We focused our transcriptome analyses on transcription factors (TF) which are known to control transcription levels and act as major developmental switches. 134 DE genes were detected as TF and the differential expression of each of these TF within tissues was also compared (Fig. 5d). Of these TFs, compared to other tissues, 21 are found to be largely upregulated in the integument (Table S2) and there are 97 down regulated transcription factors (Table S3). By comparing the results of the samples of young ovules (Fig. S10) and integument, we detected genes that are expressed throughout integument development (from early stages of the ovule to the mature integument) suggesting that there are 13 throughout integument development (Fig. 5e, Table S4).

### Differentially expressed FANTASTIC FOUR homologues

**Figure 6:**
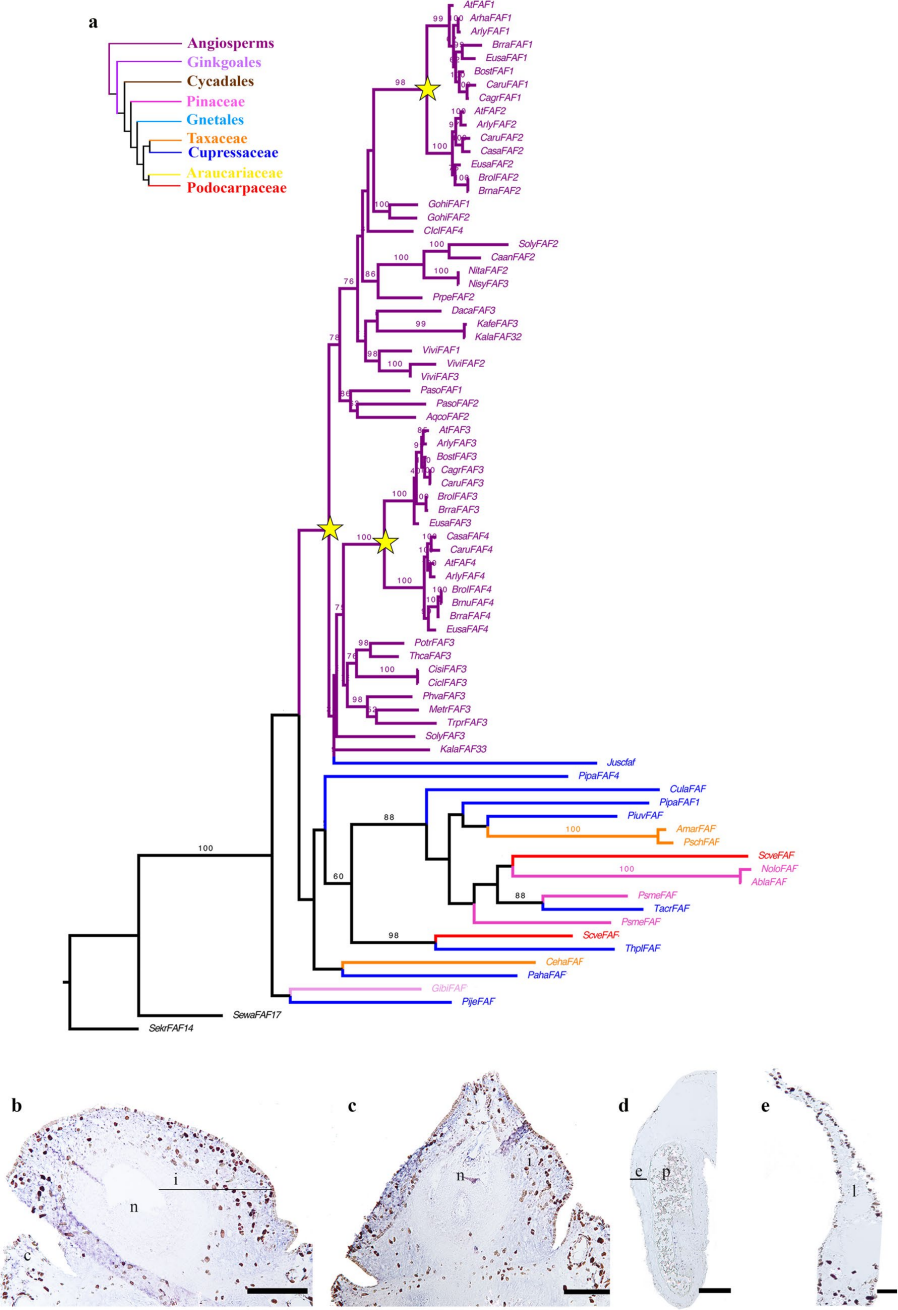
*FANTASTIC FOUR* gene family evolution and expression in *Ginkgo*. **(a)** Maximum Likelihood (ML) analysis for *FAF* homologues across seed plants. Yellow stars indicate the three large scale duplication events detected. One before the diversification of angiosperms giving rise to the clades *FAF1/2* and *FAF3/4*. And each clade has undergone one more duplication specific to Brassicaceae. **(b–e)** In situ hybridization for *GibiFAF*. **(d)** Pollen cone. **(e)** Leaf. *e* endothelium, *i* integument, *l* leaf, *n* nucellus, *p*, pollen. Scales: 75 μm (**a,b**); 100 μm (**d,e**).

Among the 21 transcription factors upregulated in the integument, the FANTASTIC FOUR (FAF) gene family stood out as they are known to repress WUSCHEL genes in *Arabidopsis*^45^. To understand the relationships among these transcripts, a detailed phylogenetic analysis of this family of transcription factors was performed. We were able to identify one sequence as a *FAF* homologue, referred herein as *GibiFAF* (Fig. 6a). We identified a duplication event occurred before the diversification of angiosperms giving rise to clades *FAF1/2* and *FAF3/4*. In addition, two Brassicaceae-specific duplication events were detected in each clade, resulting in the clades *FAF1, FAF2, FAF3* and *FAF4* respectively (Fig. 6a). With expression studies in *Ginkgo,* we found that *GibiFAF* expression is restricted to the integument throughout S4 to S9 of ovule development (Fig. 6b,c). *GibiFAF* does not appear to be expressed in the pollen cones or leaves (Fig. 6d,e).

## Discussion

Unlike angiosperms, in *Ginkgo*, we found that the expression patterns of the *WUS* homologue is not only in the nucellus but also in the integument, pollen cone, and leaf (Fig. 1a–h). In gymnosperms, the *Gnetum* homologue (*GgWUS/WOX5*)*,* exhibits expression patterns like those of monocots, in lateral organ primordia, as well as in the nucellus^48^. In the fern *Ceratopteris richardii, CrWOXB* a *WUS-RELATED* homeobox promotes cell divisions in the gametophytic generation and organ development in the sporophytic generation^53^. In land plants, all members of *WOX* gene lineage are mainly known for their function in meristem identity^20–22,54^. However, *GbWUS* expression is found in the basal region of the integuments. In *Ginkgo* ovules, the expression patterns we detected could be associated with the meristematic activity of the pachychalaza region of the ovule (Fig. 1a–f). Shifts in the expression patterns of this gene lineage in seed plants may be linked to major morphological differences or to changes in the *cis*-regulatory regions, as the protein sequence seems highly conserved in seed plants^55^.

We did not find *GibiANT* expression in young developing integuments. However, the expression pattern of *ANT* varies in ovules of different gymnosperms lineages. In gymnosperms *Pinus thumbergii,* and *Gnetum parvifolium*, expression analyses in young developing ovules show expression in the nucellus and integument^47^. In *Gnetum gnemon* and *Ginkgo* (Fig. 1m–t), expression is detected only in the micropyle at a pre-pollination stage (Fig. 1n)^13^. *ANT* in the fern *Ceratopteris richardii*, *CerANT*, is expressed in the sperm, in the archegonial neck canal just before fertilization (gametophyte structure), and in the fertilized egg, (i.e., the zygote), and in the fiddlehead (sporophyte)^56^. The expression detected in the pollen grains, suggests that *ANT* homologues were retained in gymnosperms as key factors in the development of the mega and the microspores, gametophyte development, similar to what is found in ferns (Fig. 1i,n). Overall, the ancestral function of *ANT* seems to be in cell division as it is present in active cell division regions and in young developing tissue throughout land plants^47,56^.

**Figure 7:**
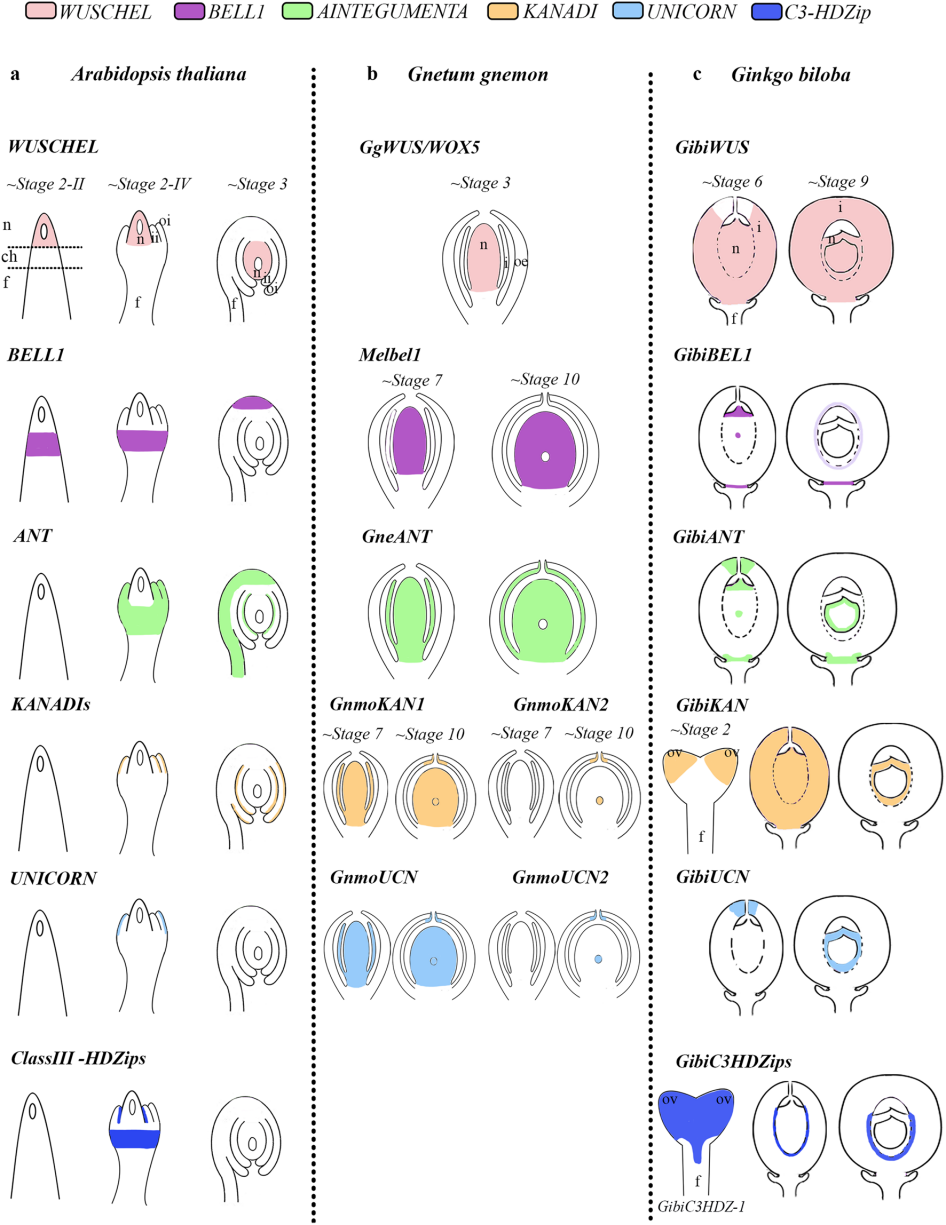
Schematic representation of the expression patterns of integument development genes, in three different species. **(a)** *Arabidopsis thaliana* (*WUS* by Grobeta-Hardt^16^; *BELL1* by Robinson-Beers et al.^58^; *ANT* by Elliot et al.^15^; *KAN* by Leon-Kloosterziel et al.^44^; Eshed et al.^40^; *UCN* by Enuguttii et al.^59^). **(b)** *Gnetum gnemon* previously published (*WUS* by Nardmannn et al. 2009; *Melbel1*, *GneANT*, *GnmoKANs* and *GnmoUCNs* by Zumajo-Cardona and Ambrose^19^). **(c)** *Ginkgo* results presented here. **(d)** Illustration of telome theory, synangial hypothesis and neo-synangial hypothesis for the origin of the seed. Notably, the telome theory indicates the evolution of integuments from sterile structures while both the synangial and neo-synangial hypotheses indicate the evolution of integuments from fertile (sporangia) structures.

In *Ginkgo*, we found *GibiBEL1* and *GibiBEL1-2* expressed in megaspore and pollen grains (Fig. 2) similar to expression of the only *Gnetum gnemon* homologue, *Melbel1*, detected in the nucellus^13^. Loss of function of *PpBELL1* in *Physcomitrella patens* moss generates bigger egg cells unable to form embryos, suggesting that *BELL1* has been key in facilitating the diversification of land plants (embryophytes^57^). This suggests that *BEL* function in the proper formation of the spores, may be conserved in mosses and gymnosperms (Fig. 2)^57^. Notably, *BEL1* in *Ginkgo* and *Gnetum gnemon* have distinct expression patterns in the nucellus (Figs. 2b–d, 7)^13^. These results allow us to infer that the function of *BEL1* homologues in the development of the egg cell is probably conserved in early land plants: bryophytes and gymnosperms (Fig. 2)^57^. However, this function does not seem to be conserved in angiosperms, suggesting major changes in the functional evolution of the *BELL1* gene lineage have occurred, following a duplication event before the diversification of angiosperms^50^. Interestingly, there are complementary expression patterns of *GibiBEL1* in the distal region and *GibiWUS* in the proximal region of the nucellus at S8 (Figs. 1d, 2e).

We did not find any polar (abaxial) expression of *GibiKAN* in *Ginkgo* ovules in particular (Fig. 3a–h). *KAN* genes are expressed in the micropylar region of the integument in gymnosperms, suggesting differences in the proximal–distal development of these ovules compared to angiosperms (Fig. 3a–h)^13^. In the lycophyte *Selaginella moellendorffii*, three *KAN* specific homologues are expressed throughout sporangium development^60^. The expression patterns in the megaspore are conserved between *S. moellendorffii* and gymnosperms. *KAN* genes are generally known for their function in establishing abaxial organ polarity in land plants^37,40–42^. This function is likely conserved in ferns^60^ and in monocot homologues^61,62^. This allow us to hypothesize that the ancestral function of *KAN* genes is in the development of the sporangium and that this function is conserved in lycophytes and gymnosperms.

It seems that the abaxial–adaxial polarity function is not conserved in the integument of gymnosperms as *UCN* homologues are expressed only in the nucellus and apical portion of the integument. Intriguingly, both *UCN* and *KAN* homologues in *Gnetum gnemon* and in *Ginkgo*, are expressed in the tips of the integuments which suggesting: (1) the interaction between *UCN* and *KAN* may be conserved in this region; (2) their function in gymnosperms may be more in establishing the proximal–distal axis; and (3) this indicates major developmental differences between gymnosperm and angiosperms ovules (Fig. 3i–u)^13,45^.

Interestingly, *GbC3HDZ1* and *3* are also expressed in the adaxial side of the integument, likely involved in the separation of the nucellus and integument in the pachychalazal region (Fig. 4, Supplementary Fig. S9). Notably, *GbC3HDZ1* and *3* expression is not only adaxial in the integument but also at the base of the ovule. In *Ginkgo*, previous studies revealed expression in the leaf primordia^63^ (Fig. 4g,p,y). *C3HD-Zip* homologues are expressed in the sporangia of the lycophyte *Selaginella moellendorffii* and the fern *Psilotum nudum*^49^. In vascular plants *C3HD-Zip*s are involved in vasculature development, also observed here in the stalk^49^. However, the ovules of *Ginkgo* are not vascularized (Fig. 4, Supplmentary Fig. S9). The data available so far suggests that sporangia development could be the ancestral function of this gene lineage^49^.

The main sources of diversity and changes underlying evolution are alterations in the expression of genes encoding transcriptional regulators^64^. We focused on differentially expressed (DE) genes annotated as transcription factors (Fig. 5c,d, Tables S3, S4, Fig. S8)^65^.

We have identified a gene upregulated in the integument transcriptome related to the *FANTASTIC FOUR* (*FAF*; Fig. 6a), a plant-specific gene family with four paralogues in *Arabidopsis*: *FAF1* to *4*^51^ (Table S2). FAF1 and 2 proteins, are known for their ability to regulate the size of the shoot apical meristem and expression in the embryo (Fig. S12)^51^; this function in the meristem is linked to its ability to repress WUS^51^. Our Maximum Likelihood analysis shows that there are three duplication events. One before the diversification of all angiosperms giving rise to two clades: *FAF1/2* and *FAF3/4* corresponding to a whole genome duplication (WGD) event ε^66^. In addition, there is a Brassicaceae-specific duplication event in each of these clades that corresponds to the α and β WGD events specific to Brassicales^66^. Gymnosperms are pre-duplication homologues (Fig. 6a). Our expression analyses in *Ginkgo* indicate that *GibiFAF* is expressed at higher levels in the integument (Fig. 6b,c) and neither in the pollen cone nor in the leaf (Fig. 6d,e) corroborating the analysis of DE genes (Fig. 6d). It is not yet clear whether *FAF* directly represses *WUS* in *Arabidopsis* as their expression overlaps, but, *GibiFAF* and *GibiWUS* expression only overlap in the integument of *Ginkgo* (Figs. 1, 6) suggesting that, *GibiFAF* is likely a novel regulator of integument development in *Ginkgo.* To determine if this function is conserved in other species, further studies are needed.

Beyond understanding morphological and developmental patterns of *Ginkgo* ovule, our results also provides molecular evidence on the origin of the seed.

1. Expression patterns do not appear to be wholly conserved between angiosperms and gymnosperms (Fig. 7), but the main function of the gene(s) may be conserved. The expanded expression of *GbWUS,* at base of the ovule and in the basal portion of the integument, indicates that this region has persistent meristematic function. *GbWUS* expression, additionally, provides molecular support for the interpretation of *Ginkgo* integument as pachychalazal, where the chalaza domain extends upward from base of the ovule. The expression of *GbC3HDZ1* in the adaxial basal region of the integument, indicates that its role in repressing the meristematic activity of *GbWUS*^12^ may have occurred early during the evolution of the seed.
2. *GibiFAF* expression indicates that it is a novel gene involved in pachychalazal and integument development. In Arabidopsis, *FAF* homologs are expressed in the shoot apical meristem and interact with WUS^51^. Therefore, *GibiFAF* and *GbWUS* in the integument supports this tissue as an expanded meristematic region. Further analyses in *Arabidopsis* are needed to determine the role, if any, of *FAF* homologues in *Arabidopsis* seed development.
3. A distinct apical-basal expression pattern is present in gymnosperms. In *Ginkgo* and *Gnetum, BEL1* (*GibiBEL1, GibiBEL1-2, Melbel1*) is restricted to the chalaza, while *WUS* and *C3HDZ1* are in the basal part of the integument; *ANT* is expressed transiently in the basal portion of integument; and *KAN* and *UCN* are restricted to the apical portion of the integuments, unlike what is observed in angiosperms.
4. Heterochrony may have played a key role in ovule developmental processes (Table S5)^67^. *BEL1* and *KAN* expression in *Ginkgo* and *Gnetum* ovules, are expressed comparably in the nucellus at the sporogenous stage (Fig. 7, Table S5), however, in *Gnetum*, it occurs prior to pollination, whereas in *Ginkgo* it occurs during pollination
5. Molecular analyses available in land plants show that integument genes are expressed during sporangia development (in lycophytes, ferns, *Ginkgo* and *Gnetum*) suggesting that the integument developmental network was co-opted from a sporangia development network.
6. The outcomes of these studies, together with recent molecular studies, provide additional molecular evidence supporting the synagial/neo-synangial hypotheses, by showing the expression patterns of integument genes in both micro- megasporangium and in the apical region of the integument of *Gnetum* and *Ginkgo*^12,13^. Indeed, the data available to date, suggests, that the sporangia development genes were co-opted for the development of the integument and that the integuments have evolved according to the synangial/neo-synangial hypothesis.

It is enticing to speculate that apical-basal expression patterns reflect the integumentary lobes envisioned in the neo-synangial hypothesis. With *WUS* in the base of the integument and the nucellus, it is not clear what mechanism accounts for the sterile integument. Recent reports suggest this could be due to BEL1 repression of SPL/NZZ^12^. Future studies of *SPL/NZZ* homologues in gymnosperms could provide further molecular support for the synangial/neo-synangial origin of the seed.

## Methods

### Expression analyses by in situ hybridization

The *WUSCHEL* homologue was previously identified with phylogenetic analysis^42^ (GenBank accession number: FM882128). Other homologues were identified with a BLAST amino acid search using *Arabidopsis* sequences as query (Table S6). *Ginkgo* sequences were identified from the OneKP database (Table S6; https://db.cngb.org/onekp). A BLAST search was performed in the genome as well, but no hits were retrieved (PLAZA database: https://bioinformatics.psb.ugent.be/plaza/versions/gymno-plaza/). The relationships of these sequences were previously shown with maximum likelihood analyses^13,50^. There are five homologues of C*lass III HD-Zip* (*C3HDZ*) genes in *Ginkgo*, which have been previously reported^49^. However, the synthesis of the probe for one of the paralogues, *GbC3HDZ5* was not effective; thus, we will present results for *GbC3HDZ1* to *4.*

Plant material was collected from the NYBG grounds (Accession number: 1353/97) and immediately fixed in FAA (FAA; 3.7% formaldehyde: 5% glacial acetic acid: 50% ethanol). Our characterization of the expression patterns begins around S4 of ovule development for most of these genes (i.e., *GbWUS, GibiANT, GibiBEL1-*2, *GibiUCN*, *GibiUCN2*, and *GbC3HDZ2* and *3*). This is because collection of ovules at early stages is highly variable as they are covered by the bracts of the short shoots. Only *GibiBEL1, GibiKAN* and *GbC3HDZ1* were assessed starting at S2. After a 4-h incubation in FAA, samples were dehydrated in a standard ethanol series, then transferred to fresh Paraplast. The samples were sectioned on a Microm HM3555 rotary microtome. DNA templates for RNA probe synthesis were obtained by PCR amplification of 280–480 bp fragments. To ensure specificity, the probe templates were designed outside of conserved domains (Fig. S2, Table S7). Sense probes were used as negative controls. The fragments were cleaned using QIAquick PCR purification Kit (Qiagen, Valencia, CA, USA). Digoxigenin labeled RNA probes were prepared using T7 polymerase (Roche, Switzerland), murine RNAse inhibitor (New England Biolabs, Ipswich, MA, USA) and RNA labeling mix (Roche, Switzerland) according to the protocol of each manufacturer. The RNA in situ hybridization was performed according to Ambrose et al.^68^. Sections were digitally photographed using a Zeiss Axioplan microscope equipped with a Nikon DXM1200C camera.

### Collection of plant material for RNAseq and extraction of total RNA from *Ginkgo*

A total of six different samples of *Ginkgo* were collected in liquid nitrogen from the NYBG grounds (Accession number: 1353/97), then processed for sequencing with three biological replicates each; thus, young ovules (ovules at S4), collar, integument, megagametophyte (from ovules at S9), pollen cone and leaf were dissected (total of 18 samples sequenced). The tissues were ground with liquid nitrogen; total RNA from these samples was extracted using QIAGEN RNeasy Kit (QUIAGEN) with a modification using extraction buffer consisting of 2% Polyvinlypolypyrrolidone (PVP, 111.14 g/mol), and 4% β-mecarptoethanol (BME; Wang et al., 2005).

### Illumina sequencing and de novo transcriptome assembly

Quality of RNA samples was assessed using Qubit^®^ 2.0 (ThermoFisher Scientific) and Agilent Technologies 2100 Bioanalyzer. Only samples with RNA Integrity Number (RIN) ≥ 8 were used to prepare sequencing libraries. RNA-Seq libraries were prepared using NEBNext Poly(A) mRNA Magnetic Isolation Module Library Prep Kit (New England Biolabs) and the resulting libraries were paired-end (PE) sequenced (2 × 150 bp) using an Illumina HiSeq2000. The average sequencing depth for each sample was ~ 40 million reads (Fig. S3).

Raw data quality was assessed using FastQC (v 0.11.5; Andrews, 2010). Sequence adapters and low-quality reads (Phred score < 5) were removed using Trimmomatic (v 0.36) with all default parameters^69^. Transcripts were assembled using AbySS (v 2.0.2)^70^ and the Trinity (v 2.8.4) software pipeline^71^ for comparison (Fig. S4). Because of better statistics, we continued to work with the Trinity assemblies (Fig. S5). An initial reference transcriptome was assembled de novo from all RNA samples and all contigs ≥ 200 nucleotides length. The quality of the transcriptome assembly was assessed based on the calculated E90N50 contig length (E90N50 ~ 1.8 Kb; Fig. S6). The initial reference transcriptome was annotated using DIAMOND (v 0.9.13)^72^. To identify possible contaminants, *Ginkgo* contigs were searched against bacterial and fungal databases mainly associated with soil and plants, sequence databases compiled from UniProt (www.uniport.org). Sequences with an identity ≥ 50% were removed from the reference transcriptome (N = 2656). This initial transcriptome was re-assembled to improve the assembly stats using AbySS^70^, the quality of the transcriptome was assessed with contig length and BUSCO annotation (Fig. S7)^73^, the resulting assembly was used for the following steps. Long open reading frames (ORF) were predicted using TransDecoder (v 3.0.0)^71^. For gene annotation, the contigs of *Ginkgo* were searched in several databases of sequence coding land plant proteins (*Amborella trichopoda*: AMTR1.0_13333, *Arabidopsis thaliana*: TAIR10_3702, *Capsicum annuum*: ASM51225v2, *Ginkgo biloba*: NCBI:txid3311, *Gnetum montanum*: NCBI:txid3381, *Oryza sativa*: IRGSP-1.0, *Picea abies*: NCBI:txid3329, *Selaginella moellendorfii*: v1.0_88036, *Vitis vinifera*: 12X_29760; available through Ensembl and Plaza for gymnosperms; Table 1).

To interpret the overall structure of these samples in terms of the gene expression, a Principal Component Analysis (PCA) was performed using the normalized TPM values, as it allows to better interpret the variation of high-dimensional interrelated dataset (with high number of variables) and to detect major differences between samples. PCA was performed using the Python packages: sklearn, seaborn, and bioinfokit (v 2.0.2) Thus, to better understand the similarities within samples a dendrogram was obtained by performing a hierarchical clustering of the samples using a ‘complete linkage’ method (Fig. S8). Dendrogram was obtained using the SciPy package on Python (v 1.5.0).

### Transcriptome abundance (RSEM) and expression level (EBSeq) analyses

These analyses were carried out following the pipeline previously proposed^74^. Sequence reads from the different plant tissues were aligned to the reference transcriptome using Bowtie2 (v 2.4.2)^75^ and RSEM (RNA-Seq by Expectation Maximization; v 1.3.0) was used to obtain estimates of transcripts abundance for all transcripts^76^. The resulting expression levels are calculated in terms of Transcripts Per Million (TPM). Transcripts were considered to be differentially expressed between integuments and the other tissues, when TPM was ≥ 0.95 for at least a single tissue and the fold change (log2FC) was ≤  − 2 and ≥ 2 with an FDR p ≤ 0.05 (Fold Discovery Rate). To identify the corresponding Gene Ontology (GO) terms, the differentially expressed genes were further analyzed with Blast2GO (v 5.2.5; Fig. S9). Data analyses and results were plotted using Matplotlib v 3.4.2 and Seaborn v 0.8.1 Python libraries (Fig. S4).

Identification of *Ginkgo* homologues and maximum likelihood analyses for gene lineages of interest.

One of the genes found in the transcriptome analyses to be putatively involved in integument development in *Ginkgo* is similar to the *Arabidopsis* gene *FANTASTIC FOUR 3* (*FAF3*; AT5G19260). To reconstruct the evolution of the *FANTASTIC FOUR* gene family, we used the four *Arabidopsis* paralogues (AT4G02810, AT1G03170, AT5G19260, AT3G06020) as a query to perform an amino acid BLAST search in seed plants, using the Phytozome and OneKP databases. A total of 88 sequences were compiled and aligned using the online version of MAFFT (v 7)^77^. Three *Selaginella* sequences were used as outgroups to root the tree (LGDQ_scaffold_2012011; JKAA_scaffold_2181098; ZFGK_scaffold_2040141).

Phylogenetic analyses using the nucleotide sequences were performed with RaxML-HPC2 BlackBox^78^. The newly isolated sequence was deposited in GenBank (accession OK255713).

## Supplementary Information

Supplementary Information 1.

Supplementary Information 2.

Supplementary Information 3.

Supplementary Information 4.

Supplementary Information 5.

Supplementary Information 6.

## Data Availability

The data underlying this article are available in the GenBank Nucleotide Database with accessions provided in the methods and supplemental material. Additional data underlying this article is available upon request to the corresponding author.
