# Supplementary material for: Expression analyses in Ginkgo biloba provide new insights into the evolution and development of the seed
Source: Sci Rep. 2021 Nov 9;11:21995. doi: 10.1038/s41598-021-01483-0 (PMC8578549; doi:10.1038/s41598-021-01483-0)
Supplement: Supplementary file 1 — Supplementary Information 1. [file 41598_2021_1483_MOESM1_ESM.docx]

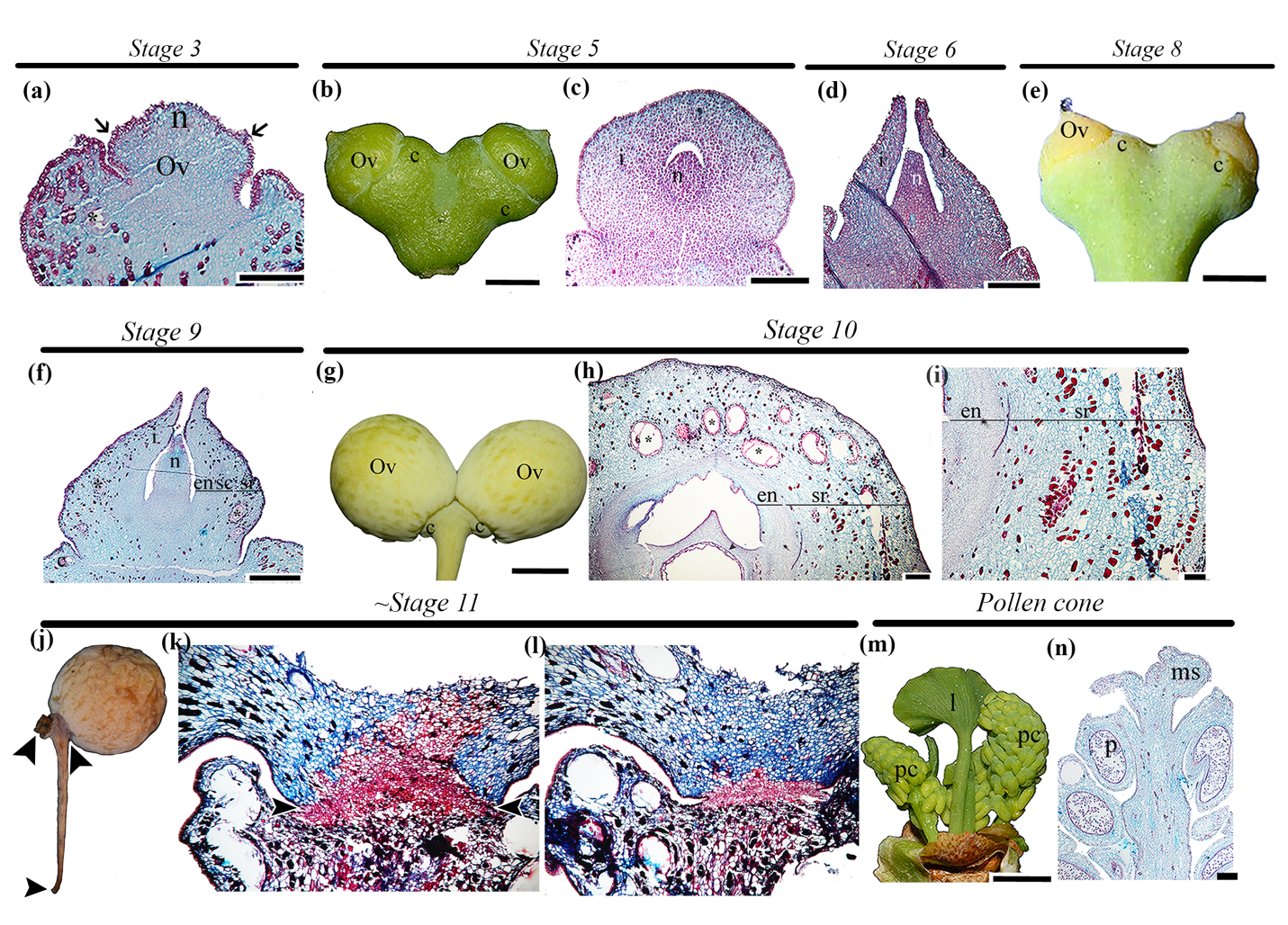


**Fig. S1.** Morpho-anatomical developmental stages of *Ginkgo* ovules. c, collar; e, endotecium; en, endotesta; i, integument; mg, megagametophyte; ms, microsporangium; n, nucellus; ov, ovule; pc, pollen chamber; sc, sclerotesta; sr, sarcotesta. *Scales*: 50μm (a); 75μm (c, d,n); 100μm (f, h-i); 1mm (b, e); 1cm (g,m).

**Fig. S3.** Number of reads obtained per sample.


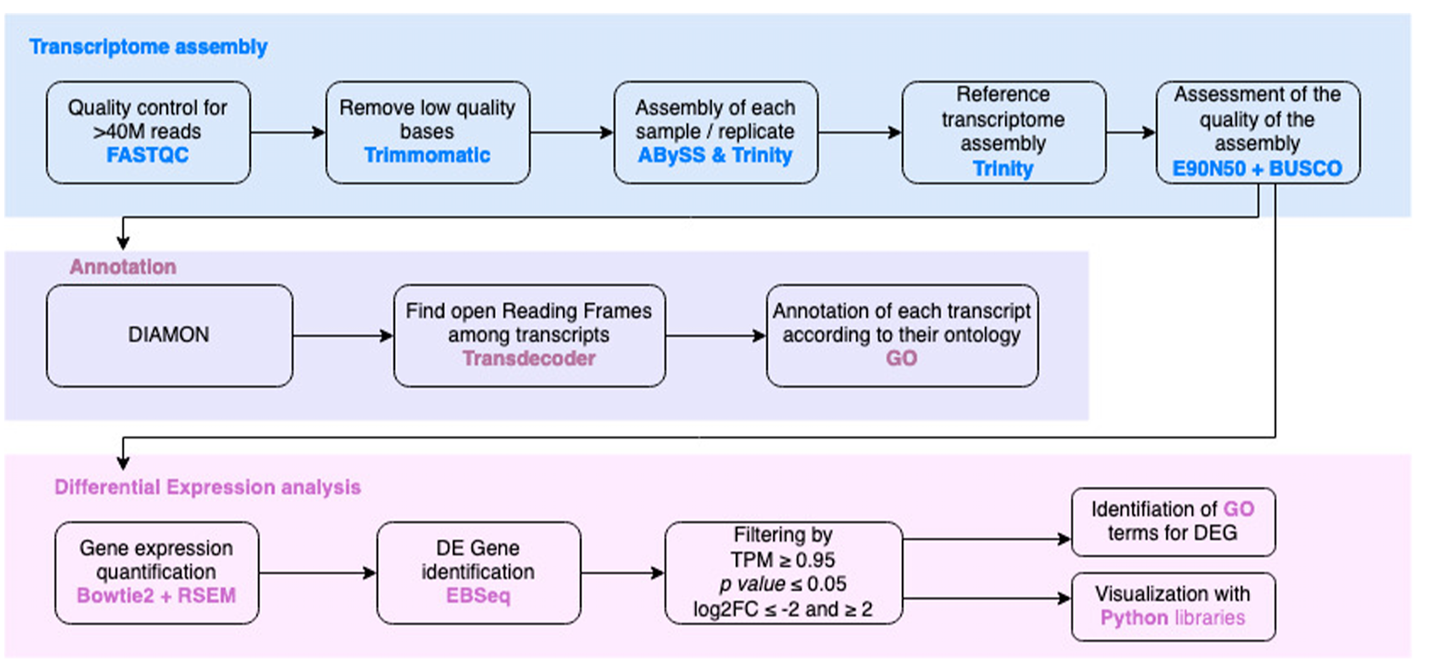


**Fig. S4.** Pipeline followed for the bioinformatic analyses.

**Fig. S5.** Statistics for the assemblies performed with Trinity and AbySS for each sample based on a weighted median statistic (N50).

**Fig. S6.** Expression-based contiguity statistics for the G. biloba reference transcriptome. The assembly has E90N50 of >1.8kb.


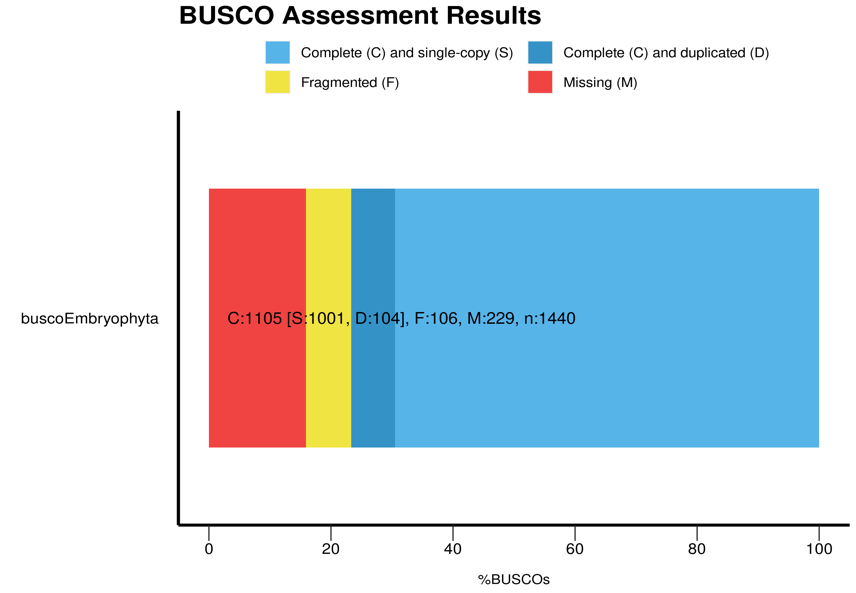

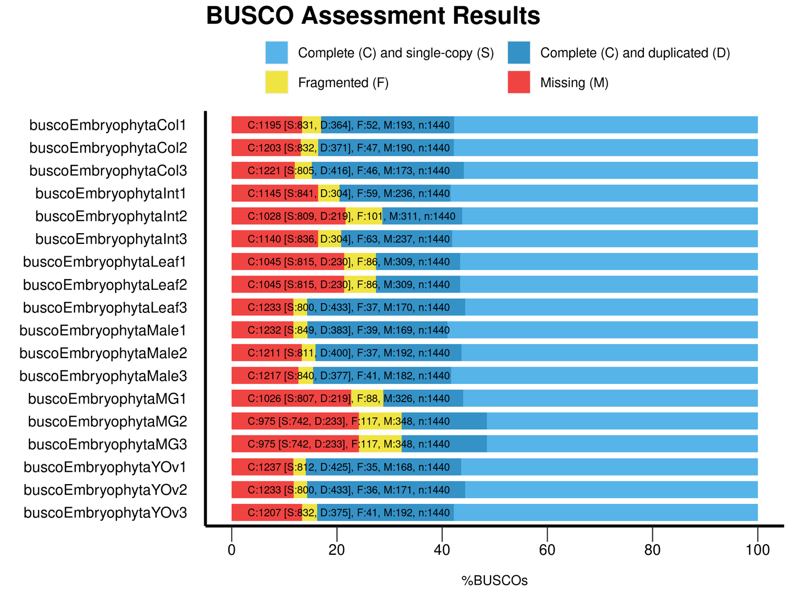


a

b

**Supplementary Figure 6. (Previous page)** Maximum Likelihood analysis of the eu*ANT* gene lineage.

**Fig. S7.** BUSCO analyses for *Ginkgo* *biloba* assemblies.

**
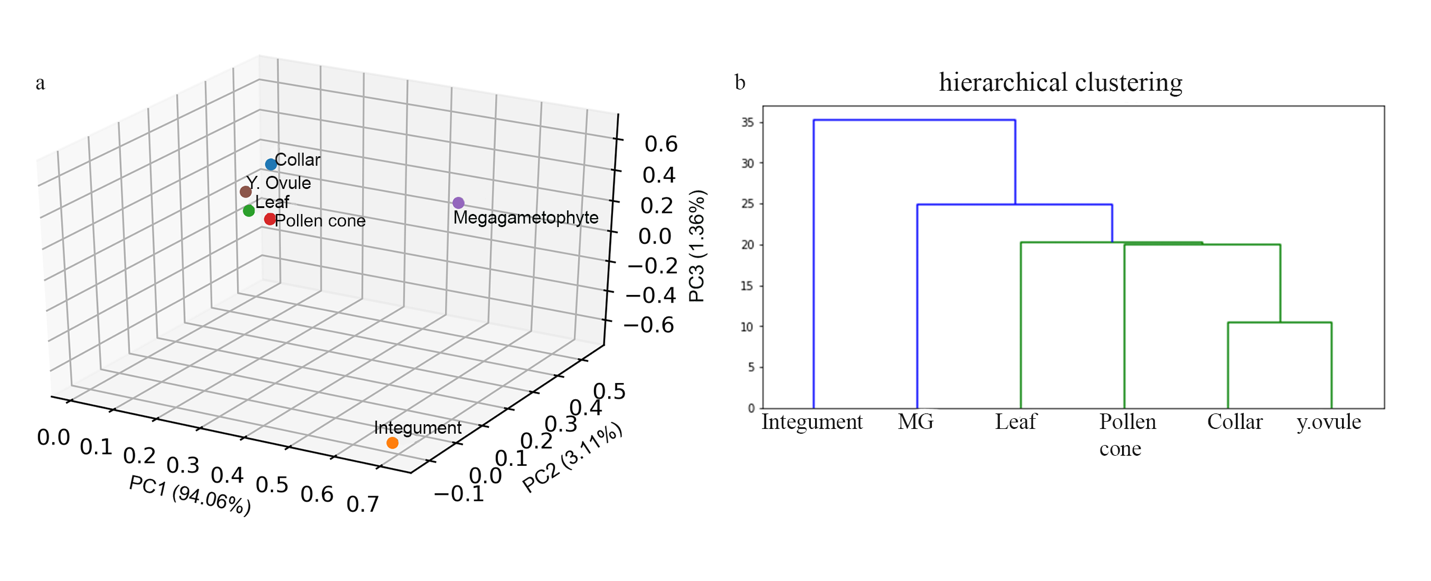
**

**Fig. S8.** Data structure **a.** Principal Component Analysis. **b.** Dendrogram by hierarchical clustering.


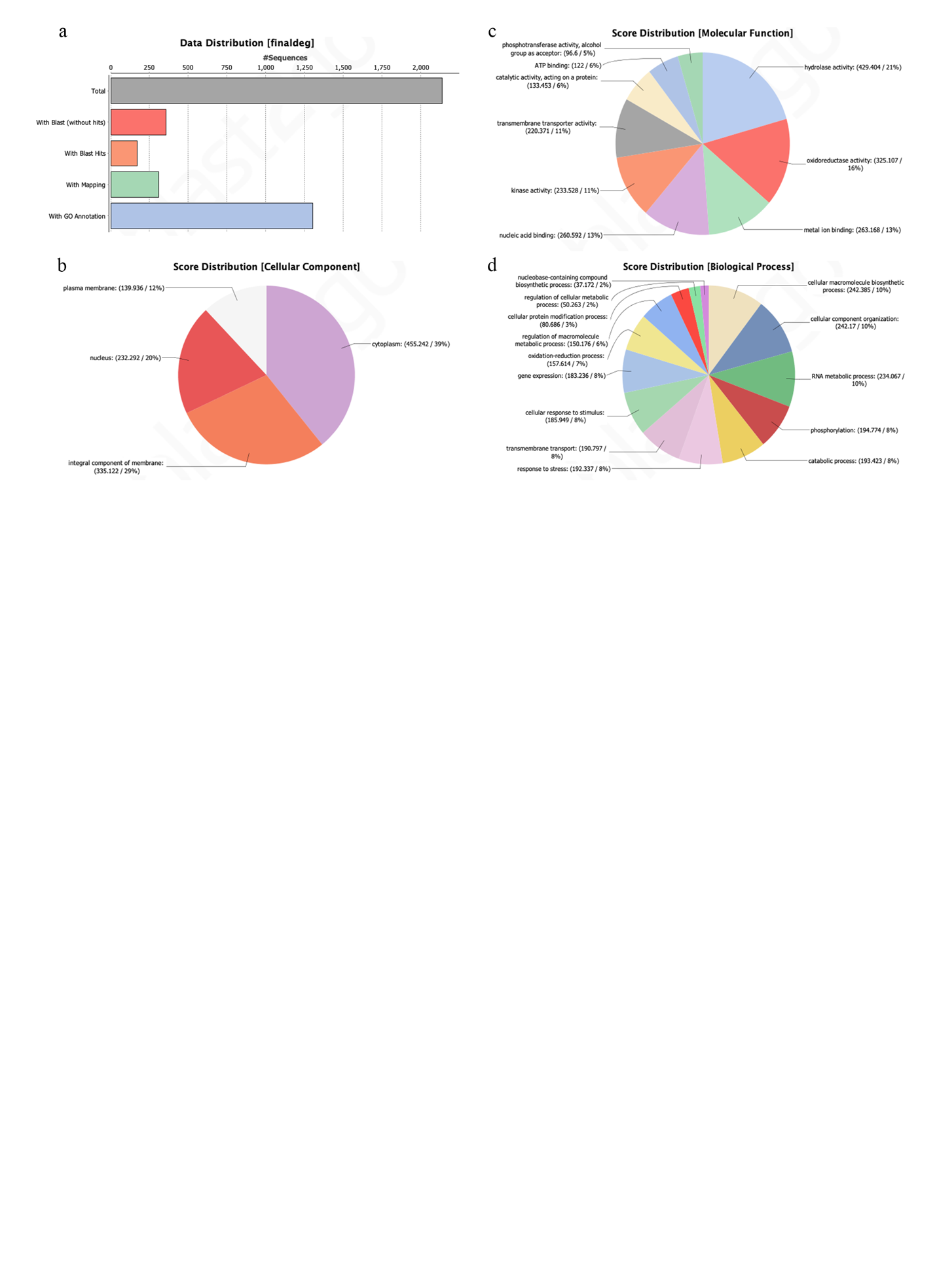


**Fig. S9.** Blast2GO statistics. **a** general statistic of the final annotation. **b** Pie chart of the Cellular component. **c** Pie chart of the Molecular function. **d** Pie chart of the Biological process.

**
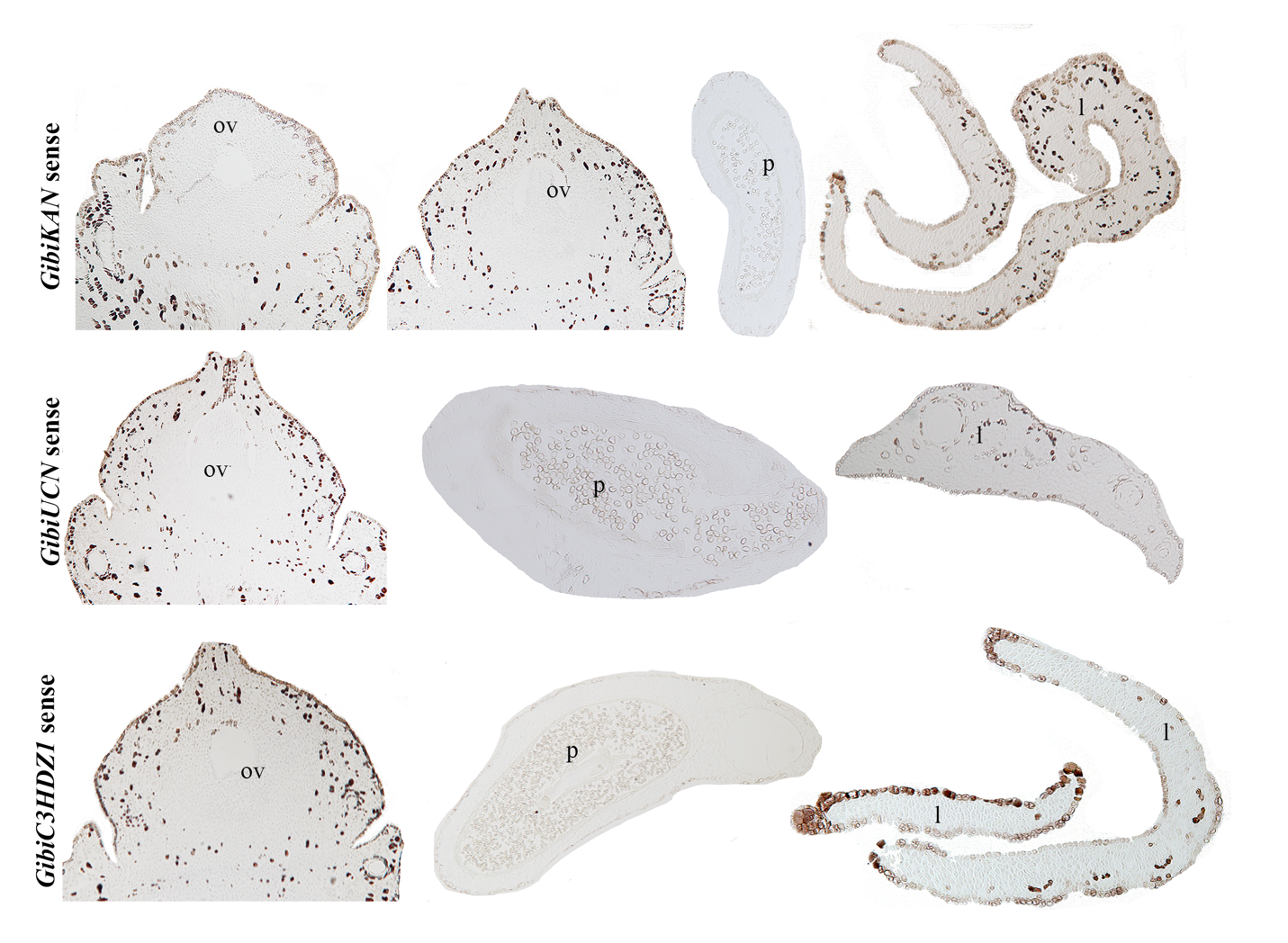
**

**Fig. S10** Sense probes in ovules (ov), pollen cones (p) and leaves (l).


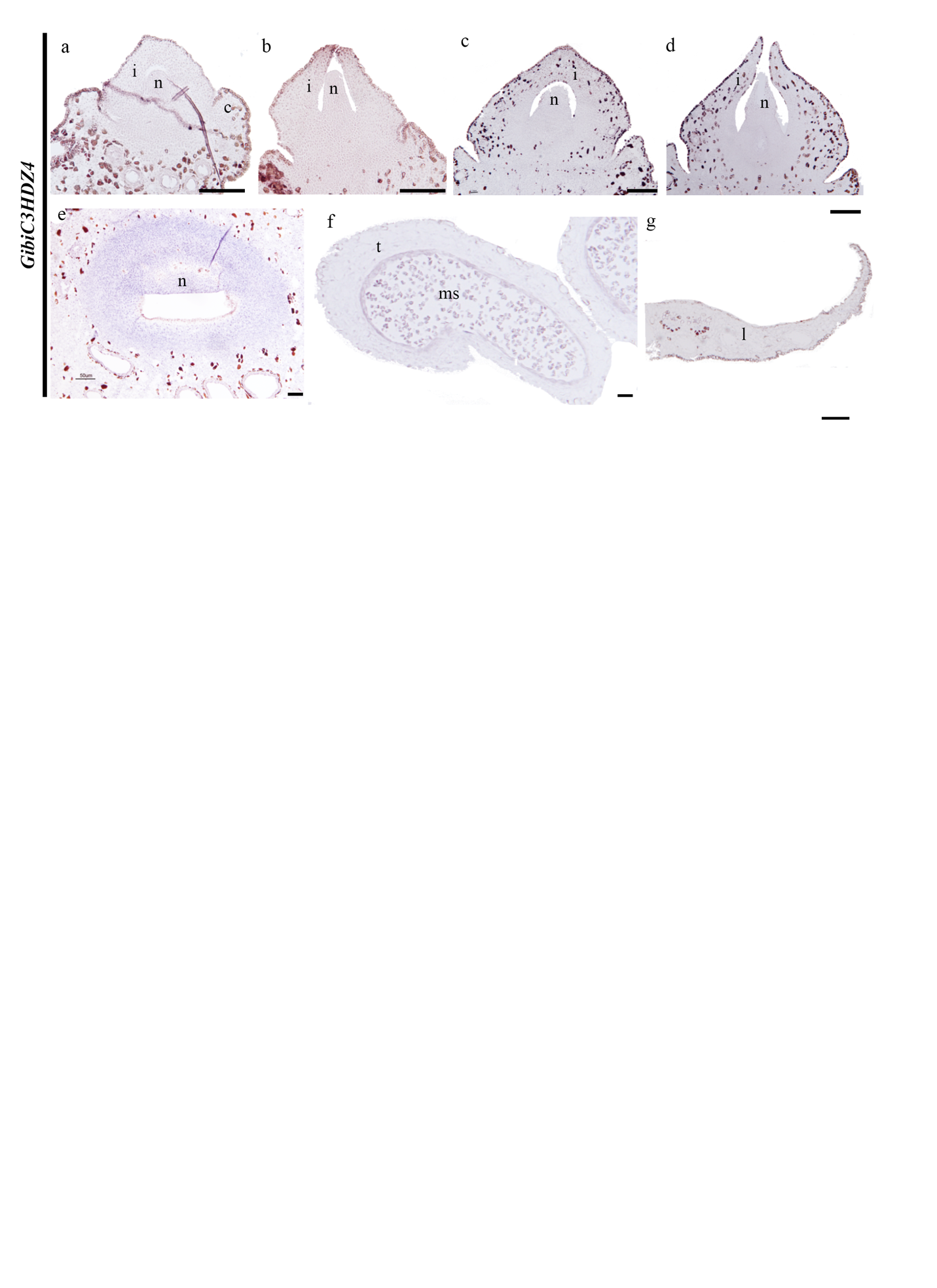


**Fig. S11.** Expression of *GbC3HDZ4* using *in situ* hybridization. **a** ovule at stage 2. **b** ovule at stage 3. **c** ovule at stage 4. **d** ovule at stage 5. **e** ovule at stage 7. **f** microsporagium. **g** cross section of a leaf.


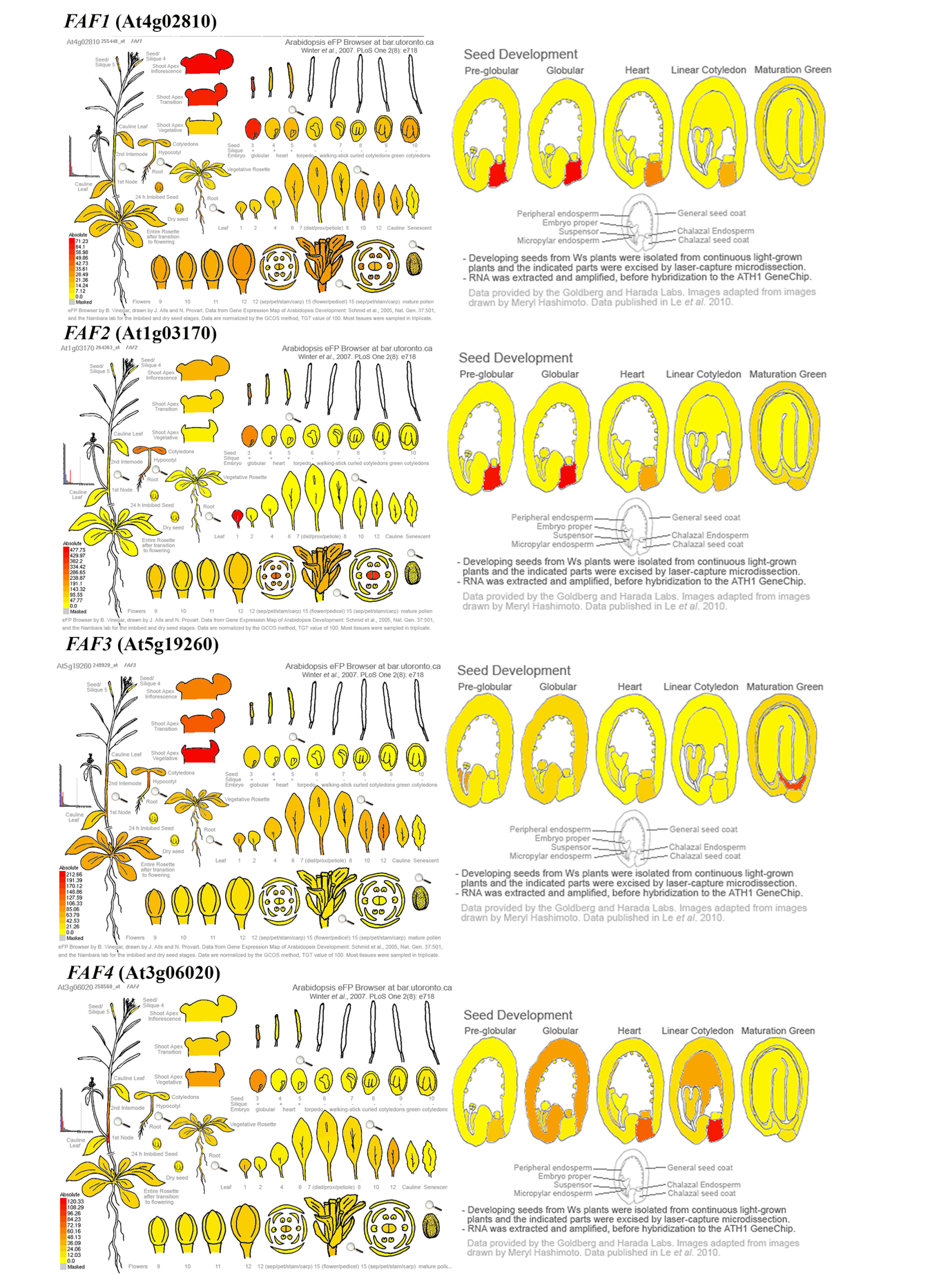


**Fig. S12.** Arabidopsis EFP browser results showing the expression of the four *FAF* paralogs on the entire plant (left) and at different seed stages (right).

**Table S1.** Comparison between the integument and all the other tissues, showing number of shared genes. This was the comparison used for the transcriptomic analyses.

|  | **Collar** | **Megagametophyte** | **Leaf** | **Pollen cone** | **Young Ovule** | **Total # of genes in Integument** |
| --- | --- | --- | --- | --- | --- | --- |
| **Integument** | 21 | 632 | 95 | 150 | 13 | 2137 |

**Table S6** *Arabidopsis* GenBank and *Ginkgo* OneKP accession numbers with the percentage of Identity between *Arabidopsis* and *Ginkgo* homologs.

| *Arabidopsis* Gene | Accession number (*Arabidopsis*) | *Ginkgo* gene name | *Ginkgo* accession number | % of ID |
| --- | --- | --- | --- | --- |
| *ANT* | AT4G37750 | *GibiANT* |  | 80.98 |
| *BEL1* | At5g41410 | *GibiBEL1* | SGTW_scaffold_2006438 | 78.74 |
| *KAN1* | At5g16560 | *GibiKAN* | SGTW_scaffold_2015986 | 83.16 |
| *KAN2* | At1g32240 |  |  | 77.66 |
| *KAN3* | At4g17695 |  |  | 81.36% |
| *KAN4* | At5g42630 |  |  | Not detected |
| *UCN* | At1g51170 | *GibiUCN* | SGTW_scaffold_2036567 | 70.95 |
|  |  | *GibiUCN2* | SGTW_scaffold_2038117 | 73.45 |

**Table S7** Primers used for *In situ* hybridization experiments in *Ginkgo biloba*

| **Gene** | **Primer sequence** |
| --- | --- |
| *GbC3HDZ1* Fwd | Ccaggttcacgagcaggag |
| *GbC3HDZ1* Rev | ccactagtaccatctgttc |
| *GbC3HDZ1*Fwd ISH(sense) | CTTAATACGACTCACTATAGGGCcaggttcacgagcaggag |
| *GbC3HDZ1* Rev ISH | CTTAATACGACTCACTATAGGGccactagtaccatctgttc |
| *GbC3HDZ2* fwd | cctgtagtgttccatcttcac |
| *GbC3HDZ2* Rev | ggagactcgagttcctgctg |
| *GbC3HDZ2* RevISH | CTTAATACGACTCACTATAGGGggagactcgagttcctgctg |
| *GbC3HDZ3* Fwd | cttcagctgcaatgaaagca |
| *GbC3HDZ3* Rev | ctaatgtacggttaggagtcg |
| *GbC3HDZ3* RevISH | CTTAATACGACTCACTATAGGGctaatgtacggttaggagtcg |
| *GbC3HDZ4* Fwd | gttcagctactttgagaactc |
| *GbC3HDZ4* Rev | gtgcaagacatgccatatcc |
| *GbC3HDZ4* RevISH | CTTAATACGACTCACTATAGGGgtgcaagacatgccatatcc |
| *GbC3HDZ5* Fwd | gtaaaacacctataagagaa |
| *GbC3HDZ5* Rev | CATTCCATGACAGAAGCTG |
| *GbC3HDZ5* Rev | gtgcgtgatagactgcacc |
| *GbC3HDZ5* Rev ISH | CTTAATACGACTCACTATAGGGCATTCCATGACAGAAGCTG |
| *GbC3HDZ5* RevISh | CTTAATACGACTCACTATAGGGgtgcgtgatagactgcacc |
| *GbC3HDZ5Fwd* | ATGCCCAGCATTATGGAGAAG |
| *GbWUS* Fwd | GGAATCCGTTCATGAGCAAGT |
| *GbWUS* Rev | TCCAGTGCCGATTCACTCC |
| *GbWUSfwd*ISH sense | CTTAATACGACTCACTATAGGGGGAATCCGTTCATGAGCAAGT |
| *GbWUS* Rev ISH | CTTAATACGACTCACTATAGGGTCCAGTGCCGATTCACTCC |
| *GibiBEL12fwd* | ctgaggactatagccttgtc |
| *GibiBEL12revISH* | CTTAATACGACTCACTATAGGGgcaggatgaactgctgaag |
| *GibiBEL12fwdISH* sense | CTTAATACGACTCACTATAGGGgcaggatgaactgctgaag |
| *GibiKAN* fwd | GGATCATCAAGGAGGCTTAT |
| *GibiKAN* rev ISH | CTTAATACGACTCACTATAGGGTGTTGGCAGGACTTGAAGC |
| *GibiKAN* FwdISH sense | CTTAATACGACTCACTATAGGGTGTTGGCAGGACTTGAAGC |
| *GibiUCN* Fwd | gacgtcttgtaaaccacaac |
| *GibiUCN* Rev | acatccaaatcagggtccttc |
| *GibiUCN* Rev ISH | CTTAATACGACTCACTATAGGGACATCCAAATCAGGGTCCTTC |
| *GibiUCN* Fwd ISH sense | CTTAATACGACTCACTATAGGGgacgtcttgtaaaccacaac |
| *GibiUCN2* Fwd | gatggcaagtgccattcattc |
| *GibiUCN2* Rev | ctttgacagacaggctcatg |
| *GibiUCN2* revISH | CTTAATACGACTCACTATAGGGctttgacagacaggctcatg |
