# Supplementary figures and images for: Expression analyses in Ginkgo biloba provide new insights into the evolution and development of the seed
Source: Sci Rep. 2021 Nov 9;11:21995. doi: 10.1038/s41598-021-01483-0 (PMC8578549; doi:10.1038/s41598-021-01483-0)

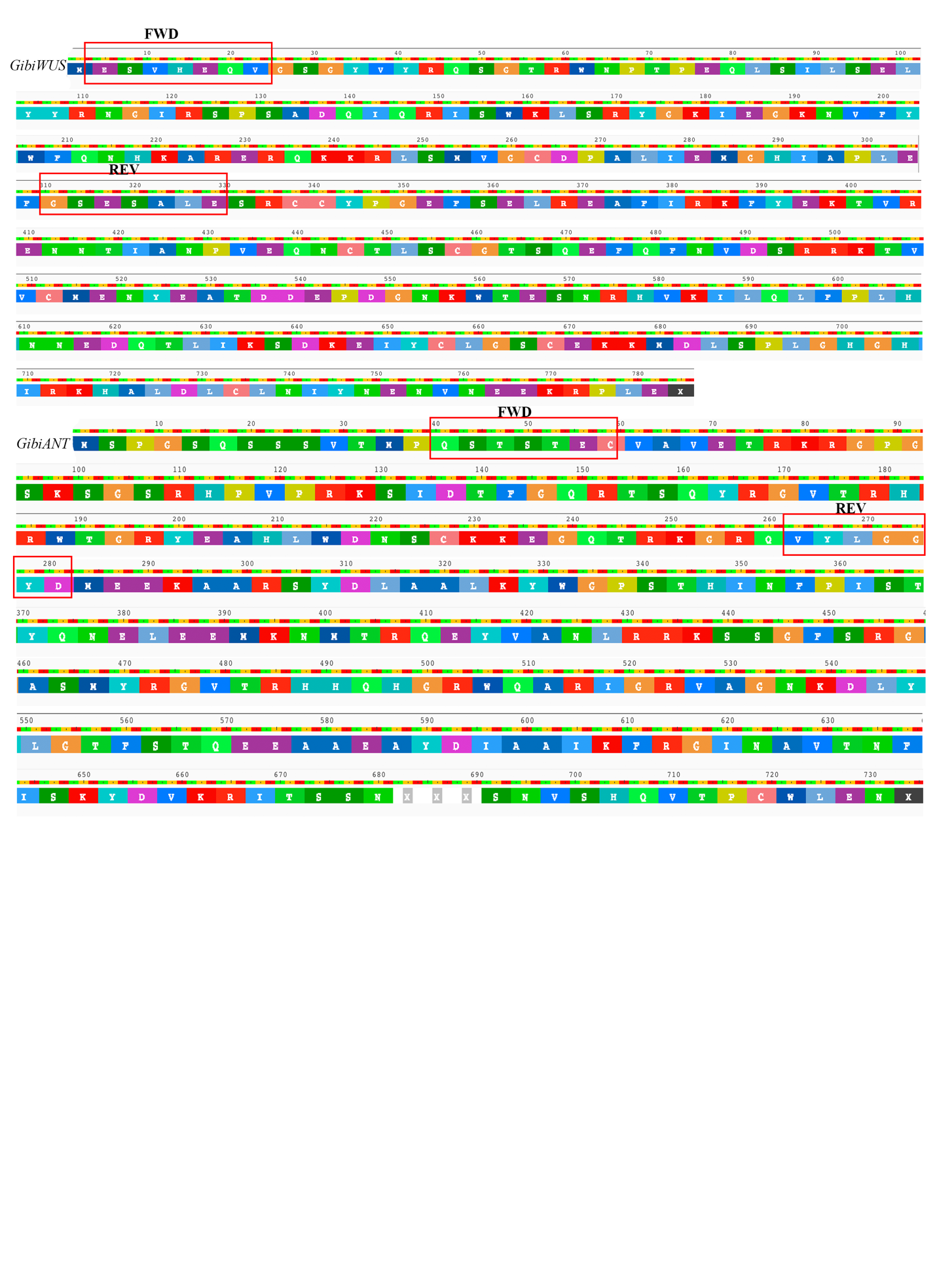


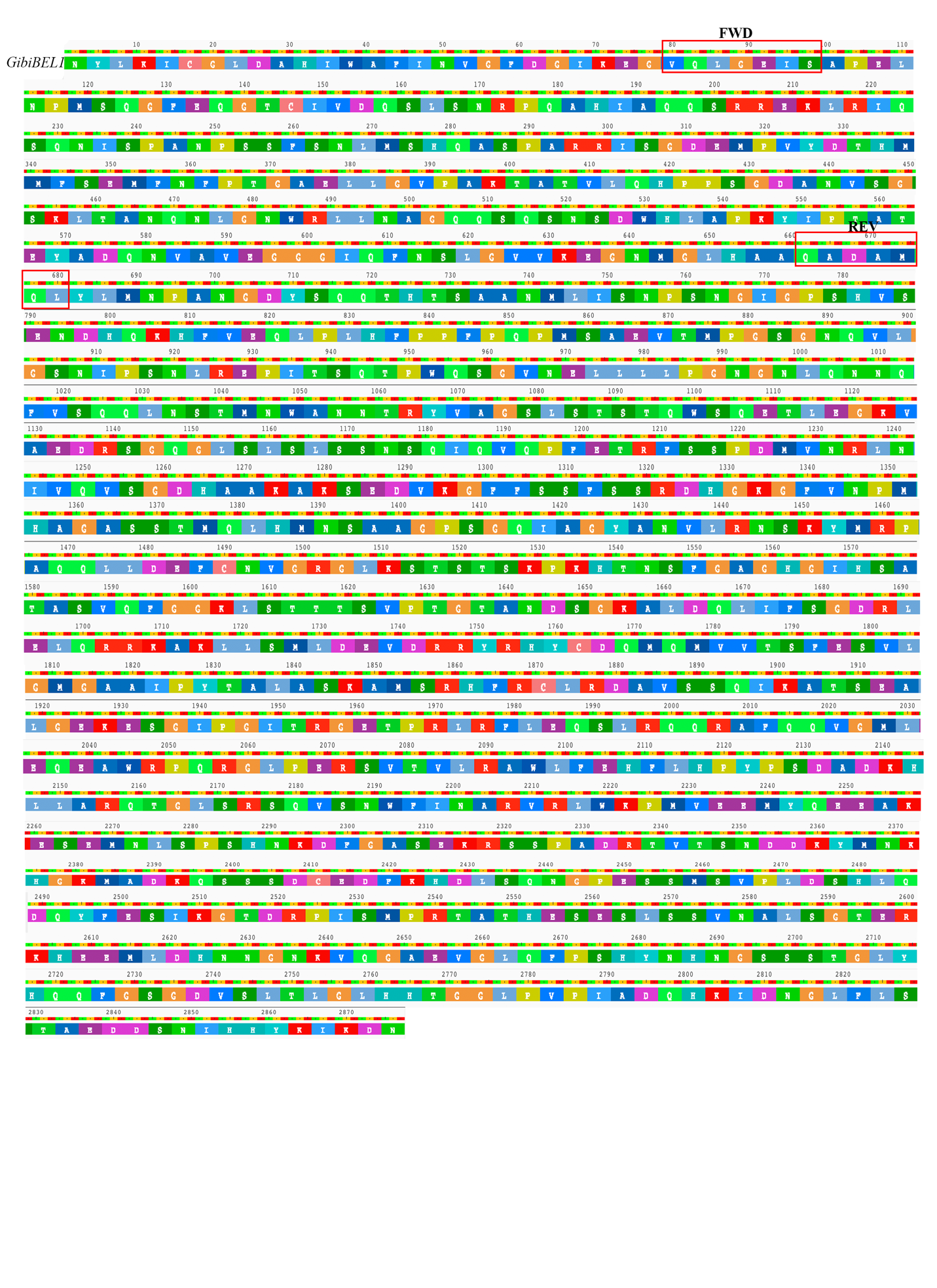


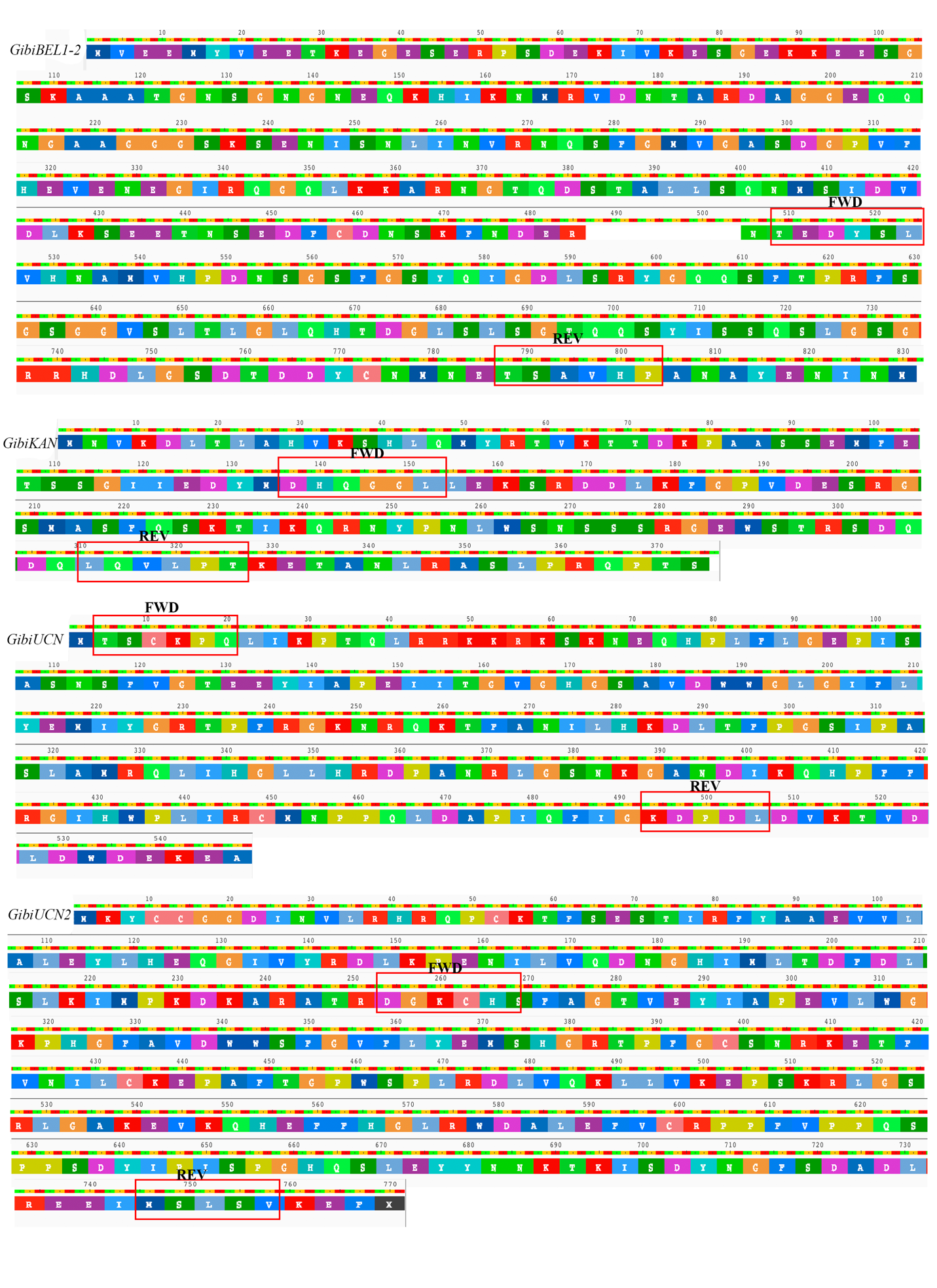


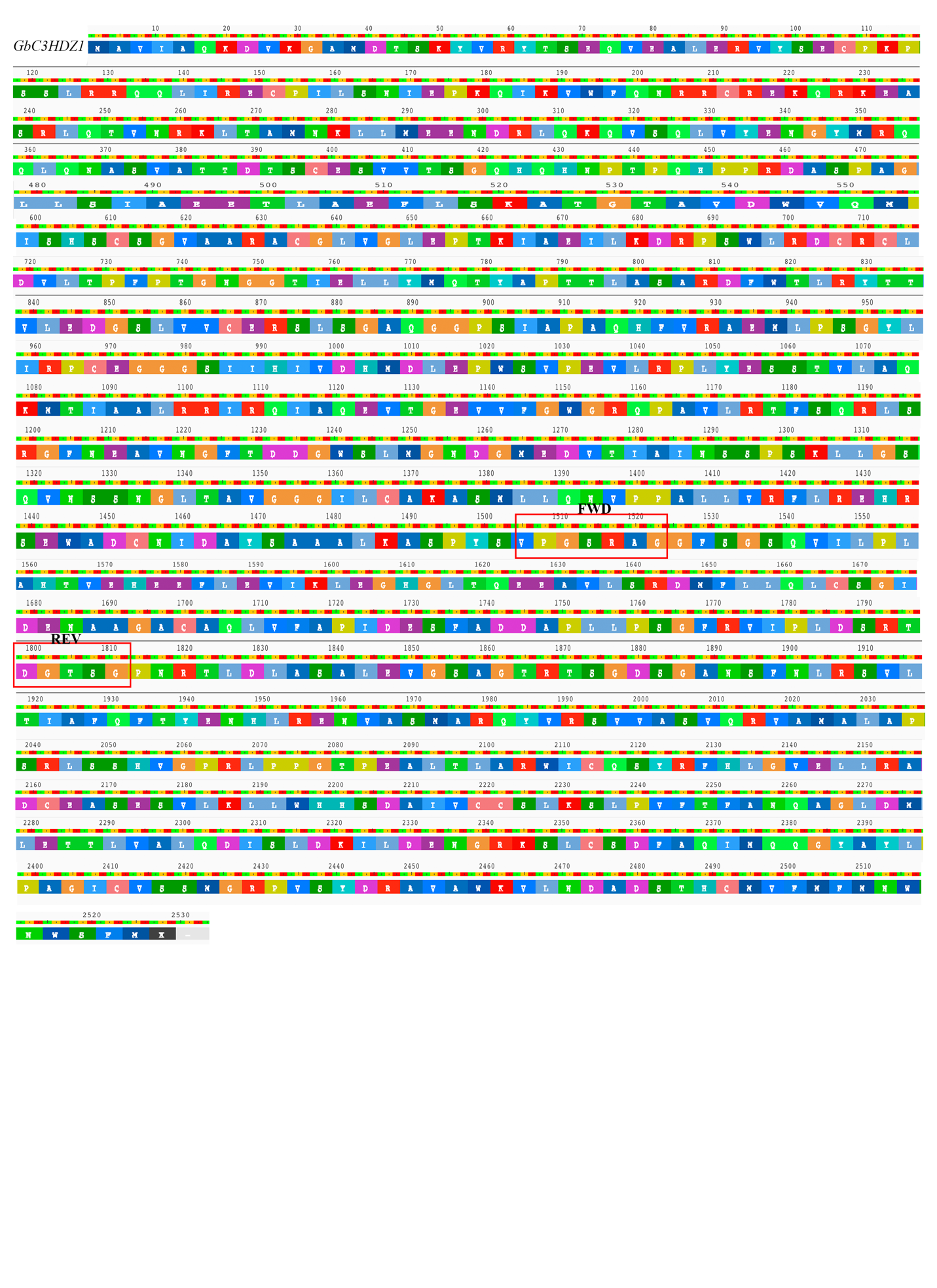


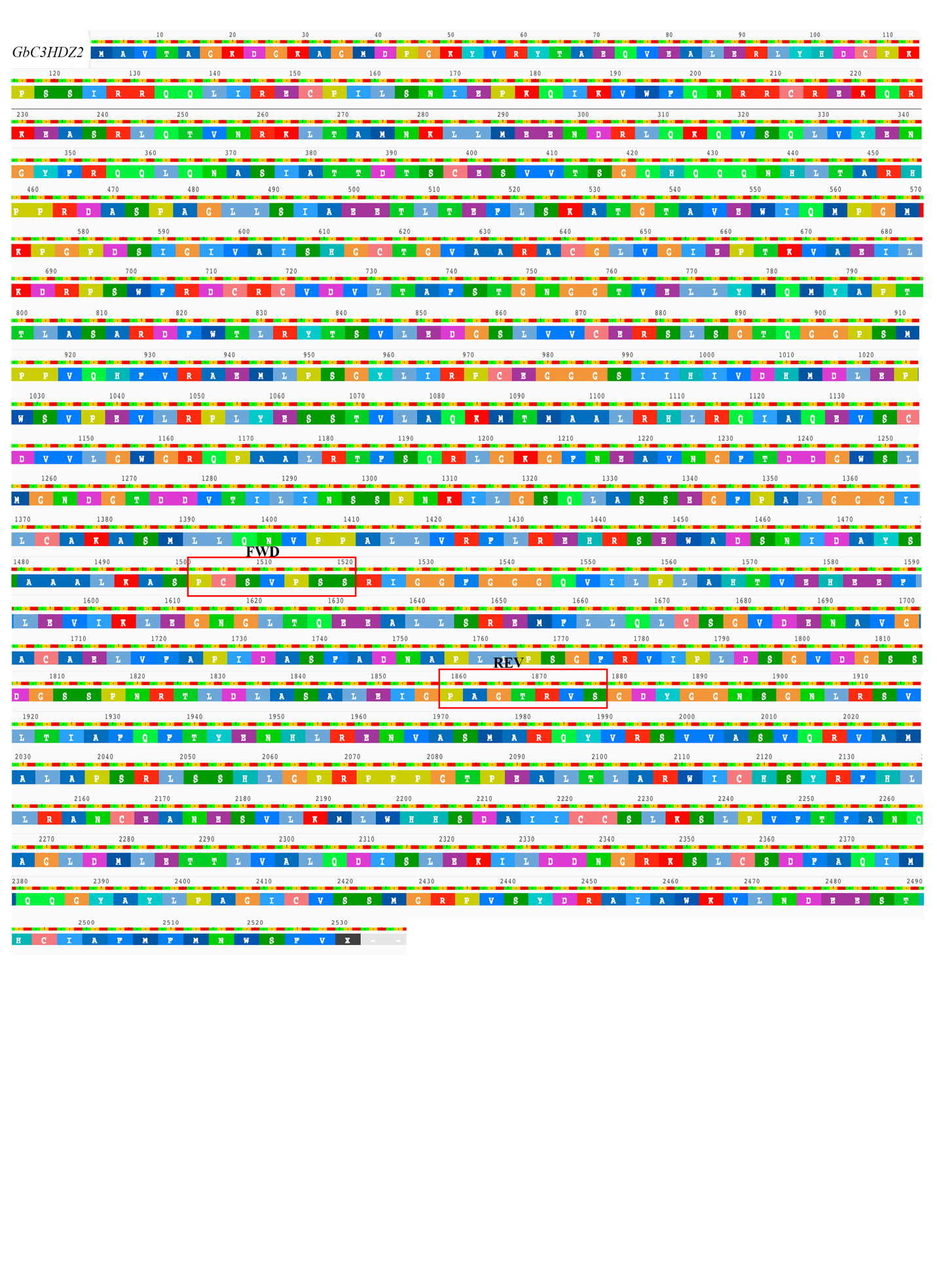


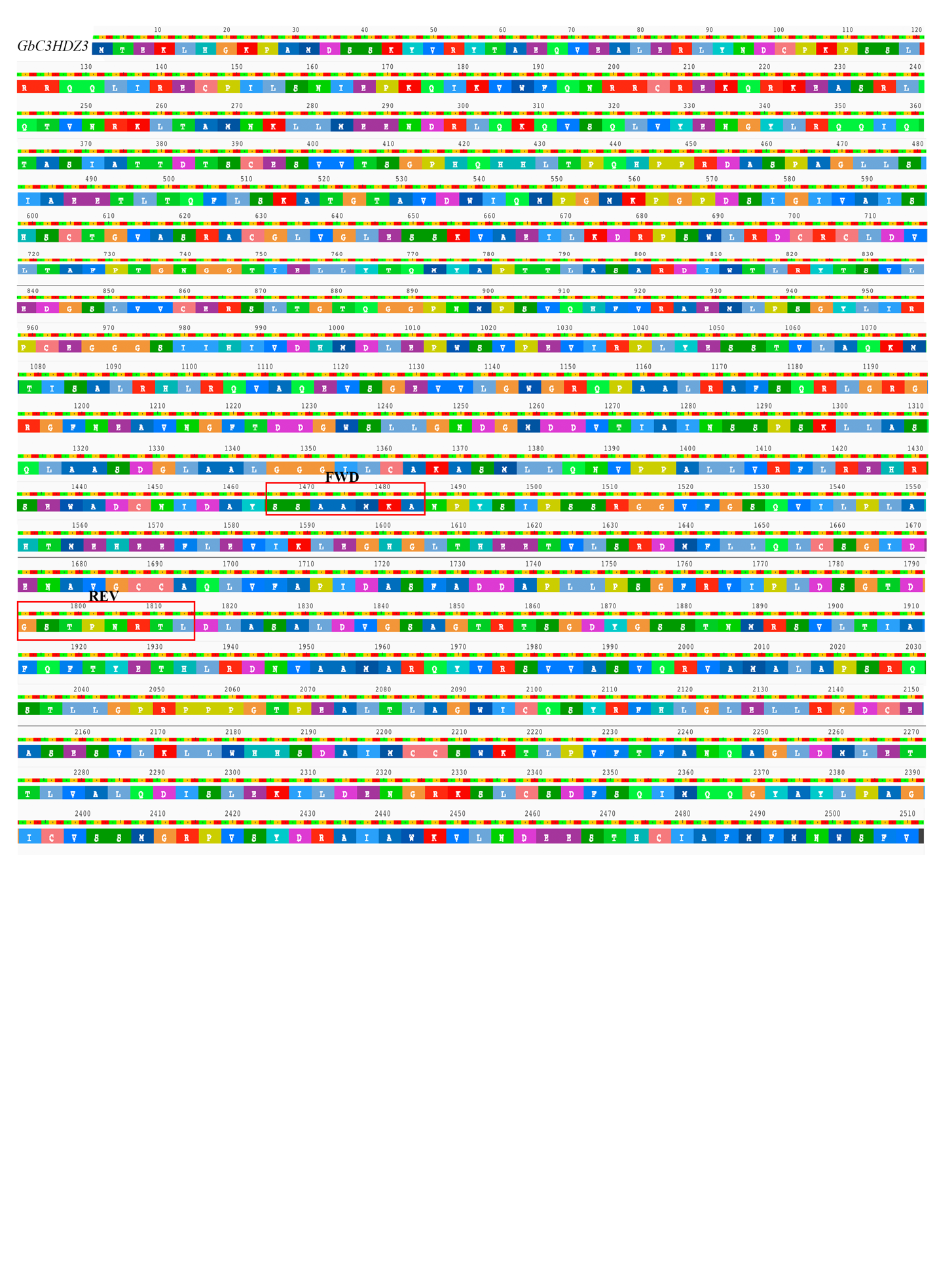


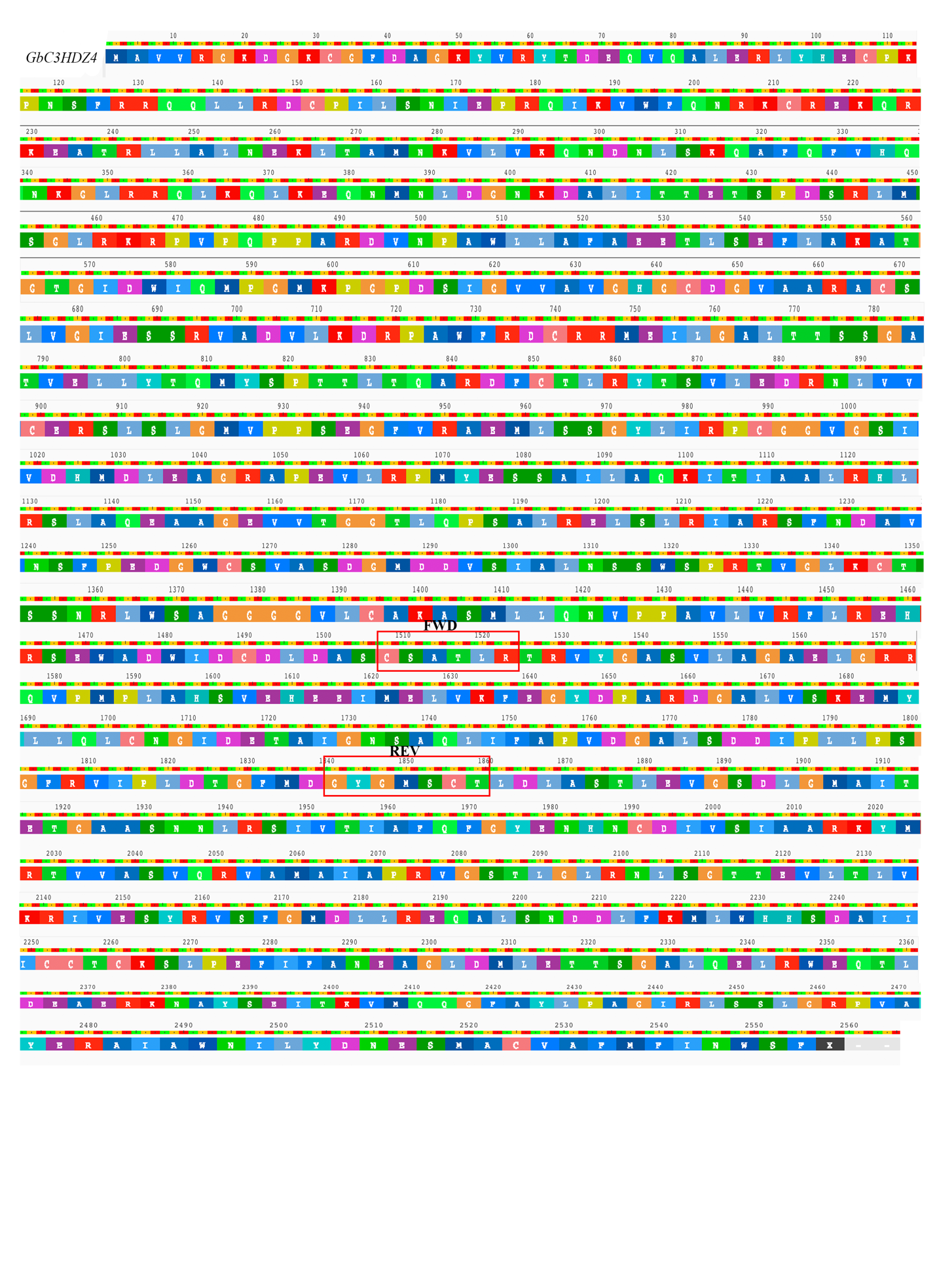

Supplement: Supplementary file 2 — Supplementary Information 2. [file 41598_2021_1483_MOESM2_ESM.docx]
