# Supplementary material for: Expression analyses in Ginkgo biloba provide new insights into the evolution and development of the seed
Source: Sci Rep. 2021 Nov 9;11:21995. doi: 10.1038/s41598-021-01483-0 (PMC8578549; doi:10.1038/s41598-021-01483-0)
Supplement: Supplementary file 6 — Supplementary Information 6. [file 41598_2021_1483_MOESM6_ESM.docx]

| ***Gnetum spp*. Ovule development landmarks.** | **Stage description (Takaso and Bouman, 1986 and Zumajo-Cardona and Ambrose, 2021)** | **Gene expression (by Nardmann et al., 2009; Zumajo-Cardona and Ambrose, 2021)** |
| --- | --- | --- |
| 1. Annular rim | Each strobilus arises in the axil of the leaf and the annular rim from which the ovules will develop is formed. |  |
| 2. Initiation of ovule primordia | 2-8 ovules (conical structure) developing from the annular rim. Dermal cells go periclinal and anticlinal cell divisions. |  |
|  |  |  |
| s3. Initiation of the outer envelope | By periclinal and anticlinal cell divisions in the dermal and subdermal cells. | *WUS* expression in the nucellus primordia (Nardmann et al., 2009) |
|  |  |  |
| 4. Initiation of the middle envelope | Initiation of the middle envelope by periclinal cell divisions. |  |
|  |  |  |
| 5. Initiation of the inner envelope (Integument) | Arises as annular primordium due to anticlinal cell div. in the middle envelope. |  |
|  |  |  |
| 6. Initial sporocyte cell divisions | As the middle and inner envelopes grow (the middle envelop does not cover the nucellus yet), several large cells in the nucellus actively divide forming parietal tissue and sporgenous cells, usually directly differentiating into a sporocyte. At this stage, the outer envelope covers the entire ovule leaving an apical cleft. | *Melbel1* in the nucellus; *GneANT* in the nucellus and integument; *GnmoKAN1* nucellus and integument; *GnmoUCN* nucellus and integument. |
|  |  |  |
| 7. Middle envelope overtops the nucellus | The middle envelope continues growing in close contact with the outer one, overtopping the nucellus. | *Melbel1* in the nucellus; *GneANT* in the nucellus and integument; *GnmoKAN1* nucellus and integument; *GnmoUCN* nucellus and integument. |
| 8. Inner envelope overtops the middle envelope | The inner envelope grows overtopping the middle envelop, reaching the length of the outer envelope. Growth occurs mostly in its apical region. Shortly before or during pollination the inner envelope starts degenerating. |  |
| 9. Pollination stage | Cells in the apical region of the nucellus form a nucellar cap. Up to 12 sporocytes are formed but only 5 remain viable dividing meiotically forming a tetrasporic female gametophyte. Note that pollination occurs at the free nucelar stage. Inner envelope forms, from outer epidermis, the 'flange' with hairs in between the outer and middle envelope. |  |
| 10. Pollen chamber | The pollen chamber forms by degeneration within the apical part of the nucellus. After pollination the whole apical part breaks off just above the top of the outer envelope. The flange from the inner envelope, closes the micropyle | *Melbel1* in the nucellus; *GneANT* in the nucellus and integument; *GnmoKAN1* nucellus and apex of the integument; *GnmoKAN2* MMC and apex of the integument; *GnmoUCN* nucellus and apex of the integument; *GnmoUCN2* MMC and apex of the integument. |
| 11. Seed development | The three envelopes remain free. The outer envelope is red and fleshy, the middle is the sclerenchymatic. In the inner envelope, the cells constituting the basal part are compressed. |  |
| ***Ginkgo biloba* Ovule development landmarks** | **Stage description (Takaso, 1980; Douglas et al. 2007; D'Apice et al., *submitted*).** | **Gene expression (Results presented here)** |
| 1. Ovule primordia | Domed shaped and smooth ovule primordia start developing. |  |
| 2. Differentiation of two ovule primordia per funiculus | Ridges and slight depressions appear on the adaxial side of the upper part of the ovulate structure, differentiating between the two ovule primordia that will be formed from the same funiculus. | *GibiKAN* in the ovule primordium |
| 3. Integument initation | The integument primordia starts developing around the nucellus as a "circular swelling". Thus, at this stage the integument and the nucellus are clearly differentiated. | *GibiKAN* in the nucellus primordium |
| 4. Integument growth covering the nucellus | The integument and nucellus continue growing but integument growth is predominant. Most cells in the ovule primordium divide anticlinal, but the apical regions of the integument and nucellus undergo primarily periclinal cell divisions. | *GibiANT* base of the ovule; GibiBEL1-2 in nucellus; GibiUCN/2 micropyle |
| 5. Integument overtops the nucellus; collar initiation | The integument grows overtopping the nucellus, with much larger cells in its apical region and more accumulation of tannins. The collar starts developing at the proximal region of the ovule, from cell divisions in the internal tissues of the funiculus. | *GbWUS* in the nucellus; *GibiANT* base of the ovule; *GibiBEL1* base of the ovule; *GibiKAN* in the nucellus |
| 6. Development of the magagametophyte | Formation of the megaspore mother cell (MMC) through meiosis. | *GbWUS* in the pachychalaza; *GibiANT* in the pachychalaza; *GibiBEL1-*2 in MMC; |
| 7. Pre-pollination stage | The integument envelops the nucellus and forms the micropyle. The apical part of the integument is lobed, at this stage the nucellus has grown up to the base of the micropyle region that will form the pollen chamber. | *GbWUS* in the pachychalaza; *GibiANT* in MMC and pollen chamber; *GibiBEL1* pollen chamber; *GibiBEL1-2* in MMC |
| 8. Pollination | Pollination drop is produced by the integument. Note that pollination occurs at the sporogenous stage (megaspore mother cell). | *GbWUS* in the pachychalaza; *GibiBEL1* pollen chamber; *GibiKAN* in the integument and nucellus |
| 9. Three Integumentary regions distinguishable | The integument differentiates into three main regions: endotesta, sclerotesta and sarcotesta (from the inside to the outside), the sarcotesta is rich in tannins and mucilage. The pollen chamber is formed to approach the pollen grains closer to the egg cell. | *GbWUS* in the pachychalaza; *GibiKAN* in the integument and nucellus; |
| 10. Formation of the Jacket cells | A three-cell layer is formed in the nucellus completely surrounding the megaspore mother cell. | *GbWUS* in the chalaza and the endotesta; GibiANT in abscission zone; *GibiBEL1-2* in jacket and abscission zone; |

**Table S2.** Summary table of *Gnetum* and *Ginkgo* ovule developmental landmarks with the corresponding expression pattern found for each of the genes of interest.
